# Epstein-Barr Virus Proteins EBNA3A and EBNA3C Together Induce Expression of the Oncogenic MicroRNA Cluster miR-221/miR-222 and Ablate Expression of Its Target p57^KIP2^

**DOI:** 10.1371/journal.ppat.1005031

**Published:** 2015-07-08

**Authors:** Quentin Bazot, Kostas Paschos, Lenka Skalska, Jens S. Kalchschmidt, Gillian A. Parker, Martin J. Allday

**Affiliations:** Molecular Virology, Department of Medicine, Imperial College London, London, United Kingdom; Tulane Health Sciences Center, UNITED STATES

## Abstract

We show that two host-encoded primary RNAs (pri-miRs) and the corresponding microRNA (miR) clusters – widely reported to have cell transformation-associated activity – are regulated by EBNA3A and EBNA3C. Utilising a variety of EBV-transformed lymphoblastoid cell lines (LCLs) carrying knockout-, revertant- or conditional-EBV recombinants, it was possible to demonstrate unambiguously that EBNA3A and EBNA3C are both required for transactivation of the oncogenic miR-221/miR-222 cluster that is expressed at high levels in multiple human tumours – including lymphoma/leukemia. ChIP, ChIP-seq, and chromosome conformation capture analyses indicate that this activation results from direct targeting of both EBV proteins to chromatin at the miR-221/miR-222 genomic locus and activation via a long-range interaction between enhancer elements and the transcription start site of a long non-coding pri-miR located 28kb upstream of the miR sequences. Reduced levels of miR-221/miR-222 produced by inactivation or deletion of EBNA3A or EBNA3C resulted in increased expression of the cyclin-dependent kinase inhibitor p57^KIP2^, a well-established target of miR-221/miR-222. MiR blocking experiments confirmed that miR-221/miR-222 target p57^KIP2^ expression in LCLs. In contrast, EBNA3A and EBNA3C are necessary to silence the tumour suppressor cluster miR-143/miR-145, but here ChIP-seq suggests that repression is probably indirect. This miR cluster is frequently down-regulated or deleted in human cancer, however, the targets in B cells are unknown. Together these data indicate that EBNA3A and EBNA3C contribute to B cell transformation by inhibiting multiple tumour suppressor proteins, not only by direct repression of protein-encoding genes, but also by the manipulation of host long non-coding pri-miRs and miRs.

## Author Summary

A relatively unbiased screen of human microRNAs (miRs) revealed that in EBV-transformed B cells, a miR cluster, miR-221/miR-222, that is frequently up-regulated in cancer, is induced by the latent EBV only if the viral nuclear proteins EBNA3A and EBNA3C are both expressed. The same two EBV proteins silence a tumour-suppressor miR cluster miR-143/miR-145. The induction of miR-221/miR-222 results from the activation of a long non-coding primary RNA (pri-miR) via long-range chromatin looping between enhancer elements that bind EBNA3A and EBNA3C and the transcription start site of the pri-miR. A well-established target of miR-221/miR-222 is the cyclin-dependent kinase (CDK) inhibitor p57^KIP2^, which, because it can inactivate various CDKs, can inhibit cell proliferation—but might have additional functions in B cells. Since EBNA3A and EBNA3C also cooperate to repress the expression of at least two other inhibitors of CDKs (p16^INK4a^ and p15^INK4b^), this implies a degree of functional redundancy in the deregulation of cell cycle checkpoints by latent EBV. This study has shown for the first time that this capacity to reduce expression of multiple cell cycle inhibitors results not only from direct repression of protein-encoding genes, but also the activation of a long non-coding RNA and cluster of oncogenic miRs.

## Introduction

Epstein-Barr virus (EBV) is a gamma-herpesvirus etiologically linked to several B cell malignancies in humans, including Burkitt lymphoma (BL), Hodgkin lymphoma (HL) and diffuse large B cell lymphoma (DLBCL). Primary infection with EBV is usually asymptomatic in early childhood, but if delayed until adolescence it may manifest as a benign lymphoproliferative syndrome known as infectious mononucleosis (IM) [1]. After primary infection the virus persists in a latent state in a memory B cell population for the lifetime of infected individuals [2,3]. Approximately 90% of the adult human population is latently infected with EBV. Moreover, *in vitro*, EBV has the unique capacity to infect, activate and induce the continuous proliferation (also known as “transformation” or “immortalisation”) of quiescent B cells leading to the establishment of lymphoblastoid cell lines (LCLs) [1,4]. These cells carry the viral genome as extrachromosomal episomes from which only nine latency-associated proteins are expressed [the latency III program—six nuclear proteins (EBNAs 1, 2, 3A, 3B, 3C, LP) and three membrane proteins (LMP1, LMP2A, LMP2B)] along with several RNA species. The latter include Bam H1 A rightward transcript (BART) RNAs that are processed to produce ~20 miRs. These latency-associated gene products act together to activate the quiescent B cells into the cell cycle and maintain their proliferation [1,4].

EBNA3A, EBNA3B and EBNA3C are three viral proteins encoded by genes that probably arose from gene duplication events during the evolution of EBV since they are adjacent in the viral genome and all have a similar gene structure—short 5’ exon and long 3’ exon [5,6]. However, despite this assumed common origin, these three proteins share only limited amino acid sequence homology and have distinct functions. Genetic studies initially indicated EBNA3A and EBNA3C, but not EBNA3B, are required and essential for B cell transformation [7,8]. Subsequently it was shown that LCLs expressing a conditionally active form of EBNA3A or EBNA3C proteins fail to proliferate when either EBNA3A or EBNA3C is non-functional [9,10]. However, more recently many LCLs have been generated using recombinant EBV from which EBNA3A has been deleted—EBNA3A-knockout (KO) viruses [11,12]. Nevertheless, EBNA3A is still believed to play an important role in B cell transformation, since cell lines deficient in EBNA3A –at least in the early stages of outgrowth—tend to exhibit reduced rates of proliferation and undergo changes in host gene expression as they become established [11,12]. In contrast to EBNA3A and EBNA3C, not only is EBNA3B unnecessary for B cell transformation in culture, *in vivo* it behaves as a tumour suppressor—apparently attenuating the oncogenic potential of EBV [13].

The EBNA3s are well established as regulators of transcription (reviewed in [14]. It seems that none of the EBNA3s bind directly to DNA and that they exert their effects on transcription through association with cellular transcription factors such as RBP-JK/CBF1, PU.1, SPI1, BATF and IRF4 [15,16,17,18,19,20,21]. EBNA3A and EBNA3C also interact with and recruit cellular factors associated with the covalent modification of histones such as histone deacetylases (HDACs), histone acetyltransferases (eg p300), CtBP and components of the polycomb group protein repressor complexes [12,22,23,24,25,26,27]. It has also recently been shown that they can regulate gene expression through the modulation of chromatin looping between distal regulatory elements and gene transcription start sites (TSS) [20]. Chromatin immunoprecipitation coupled to high throughput DNA sequence (ChIP-seq) analyses have identified many thousands of specific genomic loci where the EBNA3s can be detected—many of these sites overlap for EBNA3A and EBNA3C binding [19,20,28,29]. Probably related to this co-localisation on chromatin, independent microarray and follow-up studies revealed that EBNA3A and EBNA3C extensively cooperate in the regulation of many cellular genes [14,20,21,29]. Well characterised target genes include those encoding important survival and cell cycle regulators such as the pro-apoptotic, BH3-only protein BIM and the cyclin-dependent kinase inhibitors (CDKIs) p16^INK4a^ and p15^INK4b^ [12,27,30,31,32]. These are repressed by the combined action of EBNA3A and EBNA3C and this is probably necessary to enhance survival, prevent cell cycle arrest and inhibit cell senescence early after the infection of primary B cells by EBV (reviewed in [31]). Repression of p16^INK4A^ expression appears to be a particularly important function of EBNA3C early during the infection and transformation of B cells [30,31]. In addition to their well-established role in regulating the expression of cellular protein-encoding transcripts, we wanted to investigate whether EBNA3A and EBNA3C could also modify the expression of non-coding RNAs, particularly miRs that could also contribute to the B cell transformation process and EBV latency.

MiRs are a class of endogenous, short (~22 nucleotides), non-coding RNAs that play important roles in regulating many physiological processes including apoptosis, cell proliferation, differentiation and oncogenesis, by controlling gene expression at post-transcriptional levels. Most mammalian miRs are initially transcribed by RNA polymerase II (Pol II) that generates primary miRNA transcripts (pri-miRs), which are then cleaved by the nuclease, Drosha, into ~79-nt precursor miRNAs (pre-miRs) and exported into the cytoplasm. Once in the cytoplasm, these pre-miRNAs are processed into mature miRNAs by the Dicer nuclease and incorporated into the RNA-induced silencing complex (RISC) to target specific messenger RNAs (mRNAs) leading to either repression of translation or degradation of mRNA or often both (reviewed in [33,34]). Each miR species can generally target a large number of different mRNAs [35] and more than one species of miR can target mRNA from a single gene. There is accumulating evidence indicating that miRs are major regulators in the initiation and progression of human cancer by acting as either tumor suppressor or oncogenic miRs (oncomiRs, [36,37]). Moreover, various studies indicate that growth-transforming viruses, including EBV, can encode mimics of, and/or modulate the expression of host cell miRs (for example [4,38,39,40,41,42]).

MiR-221 and miR-222 are highly conserved, co-expressed miRs encoded as a cluster located on chromosome X and have been reported to be overexpressed in many types of cancer [43], including thyroid carcinoma [44], glioblastoma [45], prostate carcinoma [46,47], bladder cancer [48], pancreatic cancer [49], hepatocellular carcinoma [50], acute myeloid leukemia [51] and diffuse large B cell lymphoma [52,53,54]. Meta-analysis performed on over 1000 assorted human tumours, suggests that elevated expression of miR-221 and miR-222 is associated with poor overall survival of many cancer patients [55]. This well characterised oncogenic activity is likely to be related to the ability of miR-221/miR-222 to regulate cell cycle progression by directly targeting mRNA corresponding to CDKIs p57^KIP2^ (*CDKN1C*) and p27^KIP1^ (*CDKN1B*) [50,56,57,58,59].

In contrast to miR-221/miR-222, miR-143 and miR-145 are tumour suppressor miRs that have been reported to inhibit the proliferation of many cancer-and non-cancer-derived cell lines. It has also been suggested that they might play roles in cell senescence [60,61,62]. MiR-143/miR-145 coding sequences are located in a cluster on chromosome 5 and are co-transcribed as a single pri-miR transcript [61,63]. Their reduced expression has been observed in a wide range of tumours, including gastric cancer [64], colorectal cancer [65], cervical cancer [66], lung cancer [67], breast cancer [68], nasopharyngeal carcinoma [69], bladder cancer [70], prostate cancer [71] ovarian cancer [72], hepatocellular carcinoma [73] and some B cell malignancies [74]. However, although various target mRNAs have been described in these reports, none have been particularly well characterised and to our knowledge no B cell-specific targets have been described.

Here—following a relatively unbiased array screen for miRs regulated by EBNA3A and/or EBNA3C in the context of latent infection with EBV—we identified the oncogenic miR-221/miR-222 cluster as being activated and the tumor suppressor miR-143/miR-145 cluster as being repressed by EBNA3A together with EBNA3C. Further characterisation revealed that up-regulation of miR-221/miR-222 –resulting from the transactivation of a 28kb long non-coding pri-miR—was associated with almost complete ablation of p57^KIP2^ expression in EBV-infected B cells.

## Results

### Confirming leads from low-density arrays for EBNA3A and EBNA3C regulated host miRs in LCLs

In order to determine whether EBNA3A and EBNA3C regulate host cell miR levels in the context of latently infected B cells, the expression of 377 human, biologically active, mature miRs was examined using Taqman real-time PCR low density arrays (TLDA) to analyse two LCLs conditional for EBNA3C function (3CHT lines) cultured for 28 days with or without the activating ligand 4HT and two EBNA3A-KO LCLs and lines established with the respective revertant virus (REV). Several cellular miRs appeared to be regulated by either EBNA3A or EBNA3C or both. The total set of data acquired was screened for leads to be followed-up by quantitative real-time PCR (qPCR) measurements, but was not subjected to statistical analysis. Positive leads that were of particular interest—because they have been reported in the literature to have either oncogenic activity (the miR-221/miR-222 cluster) or tumour suppressor activity (the miR-143/miR-145 cluster)–were chosen for more detailed analysis. Both of these clusters are well conserved in vertebrate evolution [43,75,76].

**Fig 1:**
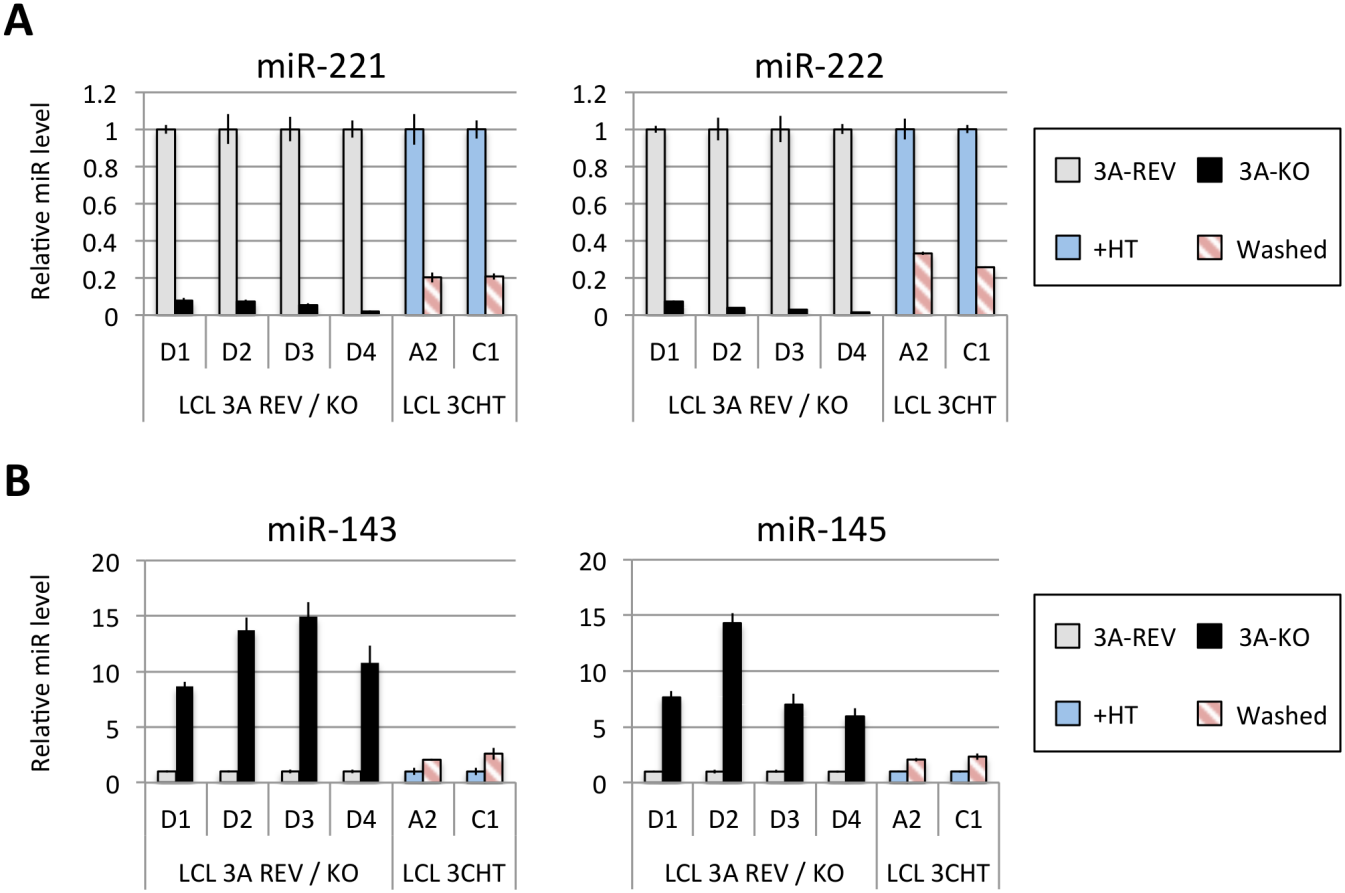
Regulation of miR clusters by EBNA3A and EBNA3C. (**A**) MiR-221 and miR-222 expression in four independent LCLs EBNA3A-KO and EBNA3A-REV (D1, D2, D3 and D4) as well as two p16-null LCL 3CHT (A2 and C1) cultured for 29 days with (+HT) or without 4HT (Washed) were determined by real time quantitative RT-PCR (qPCR). MiR-221/miR-222 expression was normalized to RNU6B and is shown relative to each “wild type” cell LCL EBNA3A-REV (3A-REV) or p16-null 3CHT cultured with 4HT (+HT). (**B**) As in (A) but analysing miR-143 and miR-145 expression.

**Fig 2:**
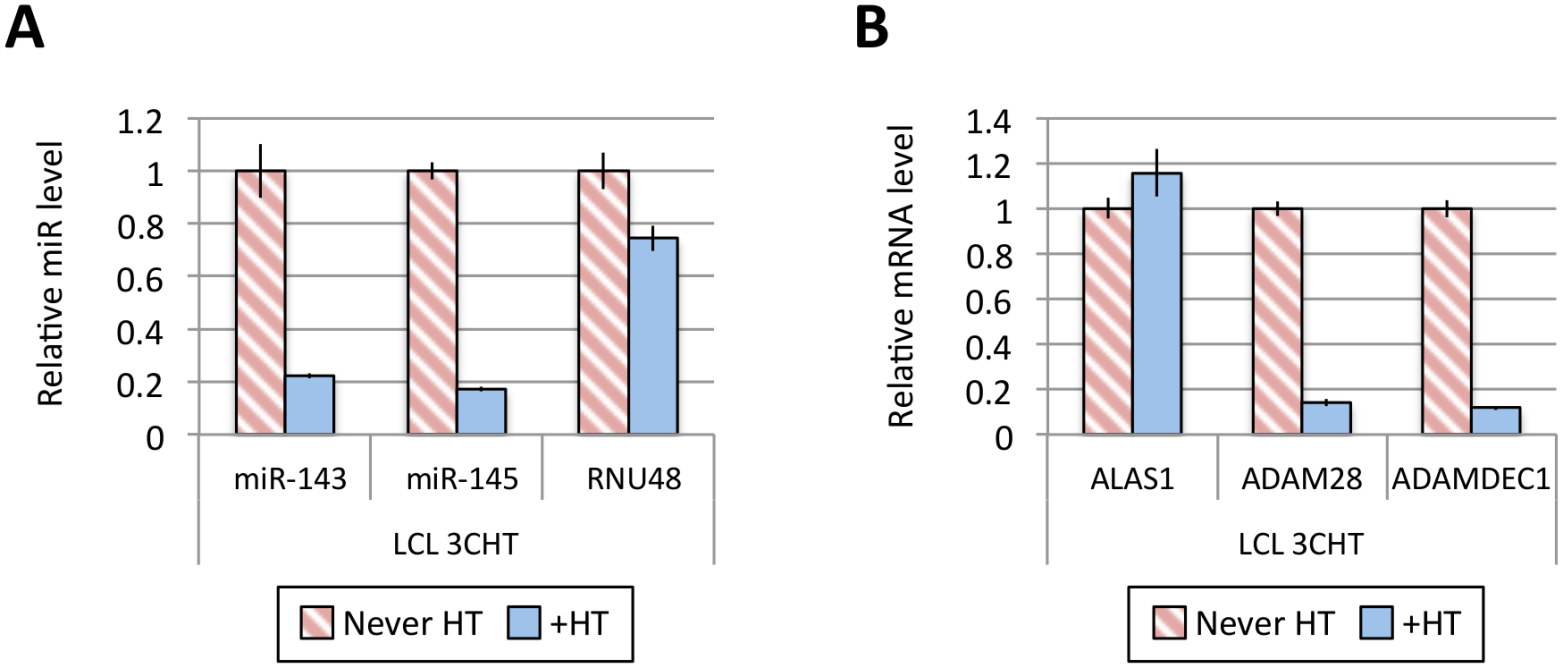
Activation of EBNA3C represses miR-143/miR-145 expression. (**A**) MiR-143, miR-145 and RNU48 expression were analysed by qPCR from p16-null LCL 3CHT established without the presence of 4HT (never HT) or 30 days after 4HT was added to culture medium (+HT). MiR levels in p16-null LCL 3CHT +HT are relative to the LCL never HT. (**B**) As in (A) but analysing the expression of ALAS1, ADAM28 and ADAMDEC1 by qPCR as controls for the activation of EBNA3C following the addition of 4HT.

Fig 1A shows the results of qPCR assays for miR-221/miR-222 in extracts from four independent EBNA3A-KO LCLs and four LCLs established with revertant viruses (and therefore expressing all the latency-associated EBV proteins). Consistently, failure to express EBNA3A resulted in a large reduction in miR-221 and miR-222 expression (Fig 1A and S1 Fig). Similarly using two independent LCLs conditional for EBNA3C function (3CHT, established in a p16-null B cell background in order to allow the cells to proliferate in the absence of EBNA3C, as described in [30]), it was shown that removal of the activating ligand (4HT) resulted in a less substantial, but clearly significant reduction in both miR-221 and miR-222 expression (Fig 1A and S1 Fig). Analysis of the same lines for expression of miR-143 and miR-145 confirmed the TLDA result showing that in the absence of EBNA3A or functional EBNA3C (by washing out 4HT) there was an increase in the expression of miR-143 and miR-145 (Fig 1B). When EBNA3A was deleted there was particularly robust expression of both miR-143 and miR-145. When conditional EBNA3C was inactivated there was a rather modest, but still significant and reproducible increase in both miRs. Consistent with this, when 4HT was added to a p16-null EBNA3C-conditional LCL that had been established in its absence (never HT), there was a substantial repression of the miR-143/miR-145 cluster (Fig 2A and S1 Fig). The differential expression of miRs are not due to the 4HT treatment since no significant change in miR expression was detected in two wild-type LCLs (D11 and D13 LCL WT) treated with 4HT for 30 days (S2 Fig). Control RNAs RNU48 and ALAS1 were unaffected by the EBNA3A or EBNA3C status of the LCLs (S3 Fig). Expression of protein-encoding gene clusters previously reported to be regulated by EBNA3A and EBNA3C (eg *CXCL9/CXCL10* and *ADAM28/ADAMDEC1*) was as expected from previous reports ([20,21,28], Fig 2B and S3 Fig). Although in some knockout and revertant LCL pairs, EBNA3B expression appeared to influence the levels of these miR clusters, the changes were very slight and/or inconsistent (S1 and S4 Figs).

### Establishment and validation of EBNA3A-ERT2 conditional LCLs

**Fig 3:**
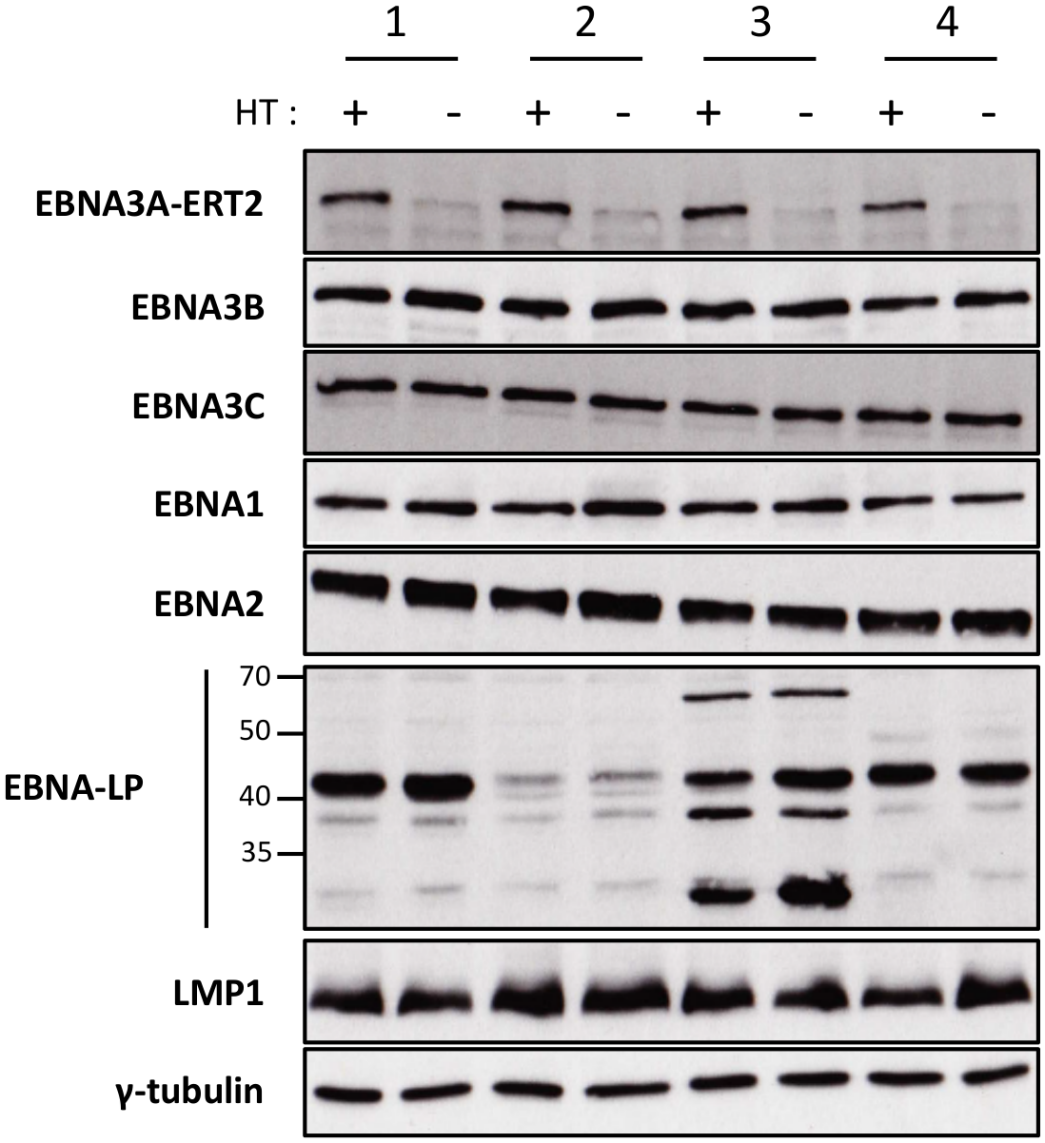
Validation of EBNA3A-ERT2 conditional LCLs. Expression of latency-associated EBV proteins EBNA1, EBNA2, EBNA3A-ERT2, EBNA3B, EBNA3C, EBNA-LP and LMP1 were analysed by Western blotting extracts from four EBNA3A-ERT2 LCLs (named 1, 2, 3 and 4) cultured in medium with 4HT (+) and 29 days without 4HT (-). The blot was probed for γ-tubulin as a control for loading.

We, and others, have found that when EBNA3A-KO LCLs are produced there is a tendency for the selection of changes in gene expression as the lines become more clonal (for example loss of/reduced retinoblastoma (Rb) expression has been reported in independent studies [11,12]). In order to establish that the changes in miR expression highlighted by the TLDA (and subsequently confirmed by qPCR) were due to direct regulation of transcription by EBNA3A –rather than the result of selection during clone development—it was necessary to construct and validate an EBV recombinant that is conditional for EBNA3A (EBNA3A-ERT2) and use this to produce new LCLs. EBNA3A-ERT2 is very similar to the EBNA3C conditional virus used in the initial assays (Fig 1 and [12,30]), but has a fusion of the C-terminus of EBNA3A with a slightly more modified estrogen-receptor that responds to 4HT but not estrogen (see Material and Methods). LCLs established with these viruses were validated for the expression of EBNA3A and other EBV latency-associated proteins by western blotting; in the absence of 4HT the EBNA3A-ERT2 fusion protein is almost completely degraded, but the expression of other latency-associated EBV proteins was unaltered (Fig 3).

### Regulation of miR-221/miR-222 and miR-143/miR-145 in EBNA3A-ERT2 conditional LCLs

**Fig 4:**
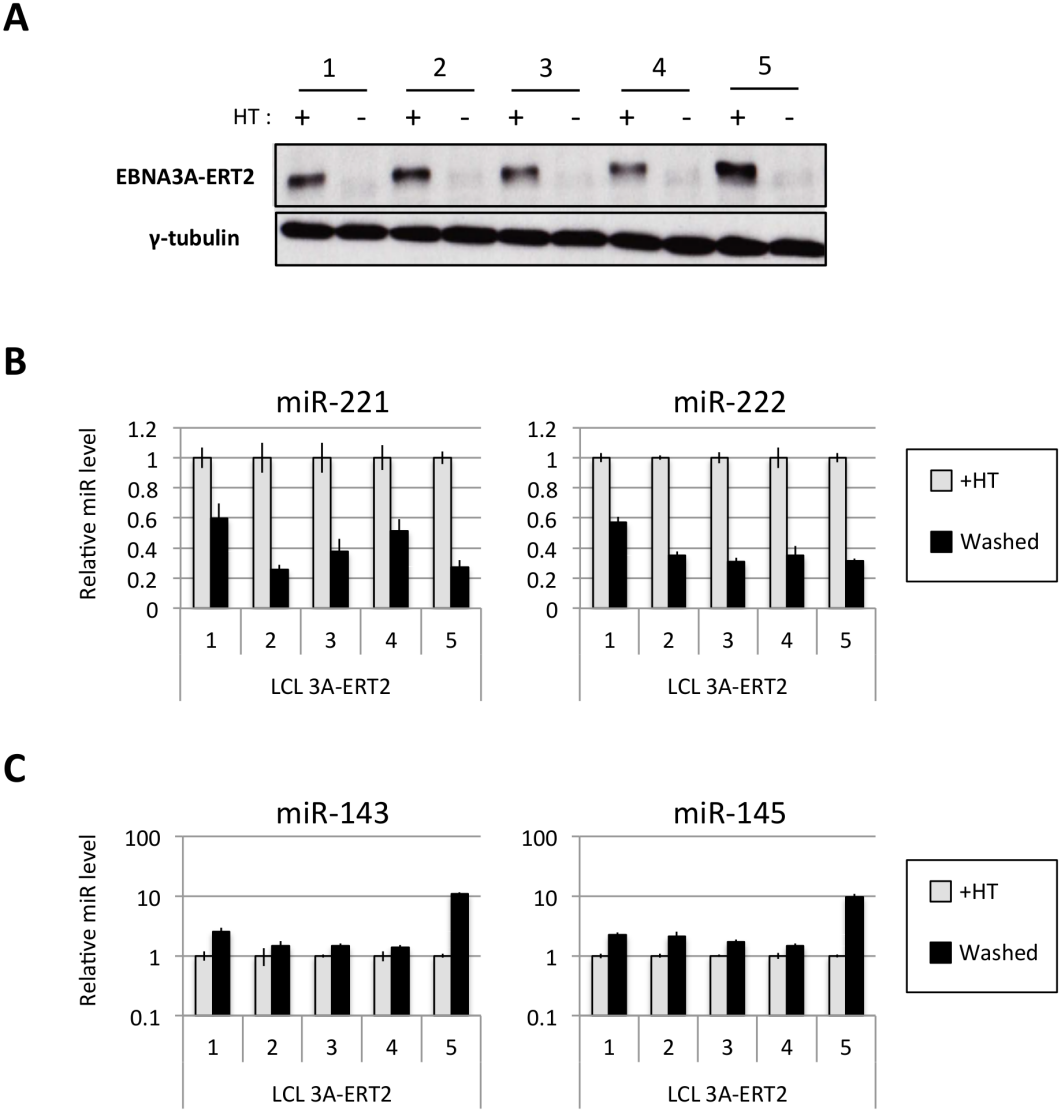
Regulation of miRs in EBNA3A-conditional LCLs. (**A**) Five EBNA3A-ERT2 LCLs (established in the presence of 4HT and named from 1 to 5) were cultured for ~30 days with (+) or without (-) 4HT and EBNA3A-ERT2 protein expression was analysed by western blot. (**B**) MiR-221/miR-222 expression was determined by qPCR using total RNA extracted from the same five EBNA3A-ERT2 cell lines (LCLs 1, 2, 3, 4 and 5). (**C**) As in (B) but analysing miR-143 and miR-145 expression.

**Fig 5:**
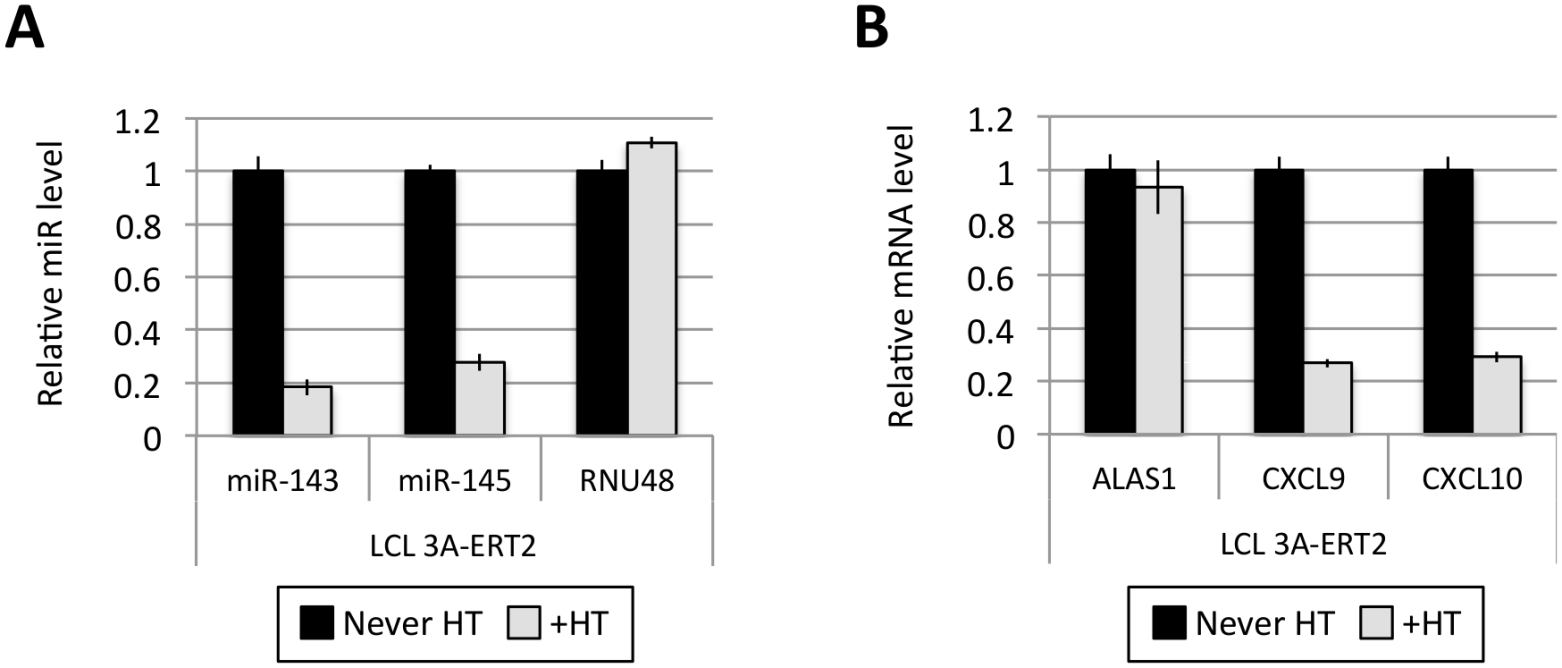
Activation of EBNA3A represses miR-143/miR-145 expression. (**A**) MiR-143/miR-145 and RNU48 expression were determined by qPCR in EBNA3A-ERT2 LCL established without the presence of 4HT (never HT) and 28 days after addition of 4HT to culture medium (+HT). (**B**) As in (A) but analysing the expression of ALAS1, CXCL9 and CXCL10 as a controls for activation of EBNA3A following the addition of 4HT.

Several EBNA3A-ERT2 LCLs produced from two different individual B cell donors (D11 for LCLs number 1–2 and D13 for cell lines number 3–4) and a mixed donor population of B cells (LCL number 5) were established in the presence of 4HT and then analyzed ~30 days after removal (-4HT) or leaving in 4HT (+4HT) (Fig 4A). Consistently on removal of 4HT (washed), miR-221 and miR-222 were expressed at a lower level (Fig 4B), whereas miR-143 and miR-145 were modestly induced (Fig 4C). As with the experiments using conditional EBNA3C, when EBNA3A-conditional cells that had been grown into LCLs in the absence of 4HT (never HT), there was a substantial repression of miR-143 and miR-145 when 4HT was added to the culture medium (Fig 5A and S1 Fig). All these experiments showed that—like EBNA3C –active EBNA3A is necessary for the regulation of both miR clusters, ruling out the possibility of clonal selection as an explanation for the changes in expression seen in the EBNA3A-KO lines. EBNA3A-ERT2 function was further validated by qPCR for expression of previously characterised EBNA3A target genes (*CXCL9* and *CXCL10* [21], Fig 5B and S5 Fig).

### EBNA3A and EBNA3C up-regulate expression of pri-miR-221/222

**Fig 6:**
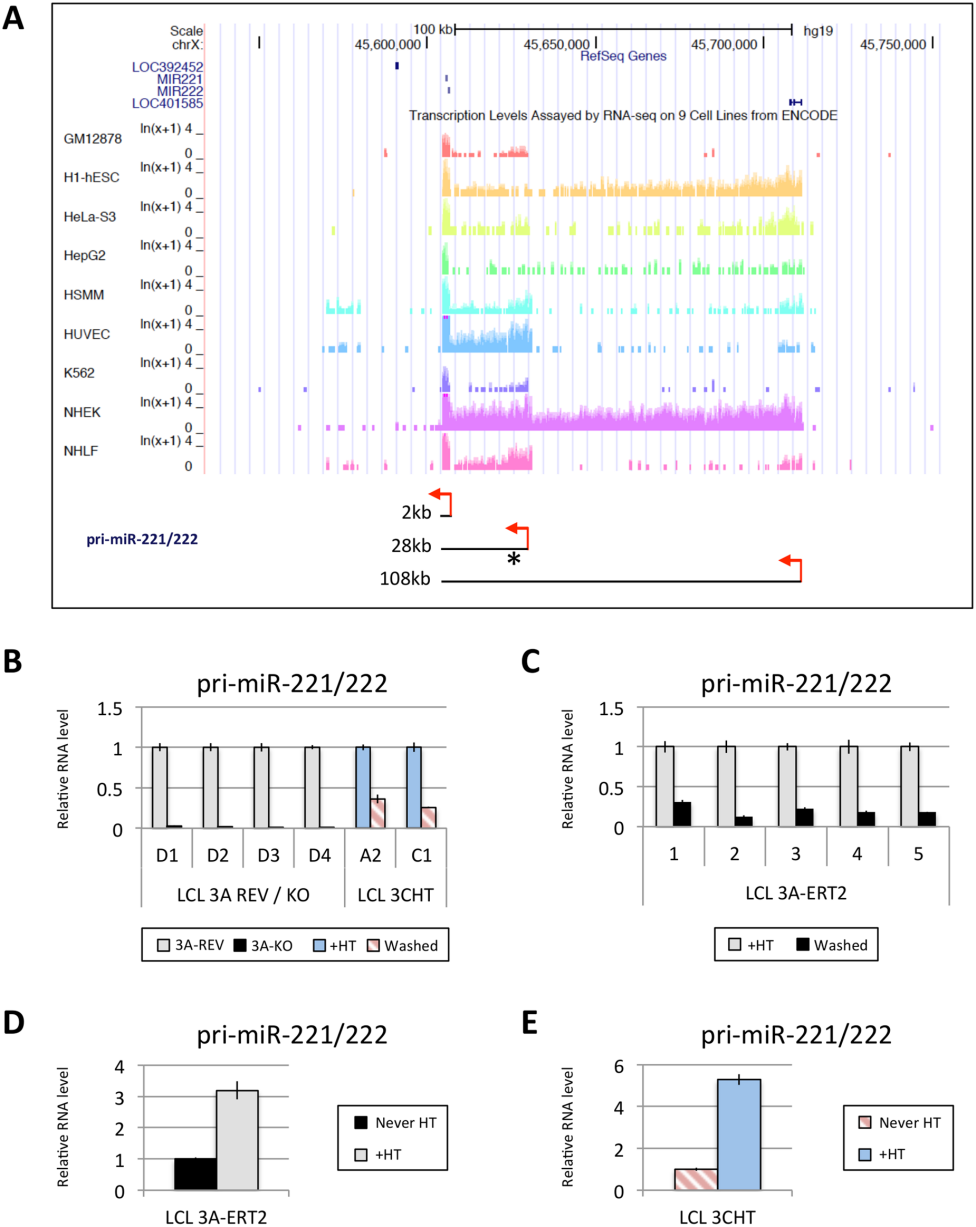
EBNA3A and EBNA3C up-regulate a pri-miR-221/222 of ~28kb. (**A**) Screen-shot of UCSC Genome Browser at miR-221/miR-222 cluster genomic locus shows the position of mature miR-221/miR-222 as well as RNA-seq data for GM12878 (LCL), h1-hESC, HeLa-S3, HepG2, HSMM, HUVEC, K562, NHEK and NHLF cell lines. These RNA-seq data from ENCODE indicate the presence of different sizes of pri-miR-221/222 between cell lines (approximately 2kb, 28kb and 108kb). Red arrows represent the proposed transcription start sites and the asterisk shows the position of primers used. The expression level of the 28kb pri-miR-221/222 was determined by qRT-PCR in (**B**) EBNA3A-KO and-REV LCLs as well as in p16-null LCLs 3CHT cultured for 29 days with (+HT) or without 4HT (Washed); (**C**) in five EBNA3A-ERT2 LCLs cultured with (+HT) or without 4HT (Washed) for ~30 days; (**D**) in LCL EBNA3A-ERT2 (never HT) cultured for 28 days with (+HT) or without 4HT; (**E**) and in p16-null LCL 3CHT (never HT) cultured for 30 days with (+HT) or without 4HT.

MiR-221 and miR-222, which together form a cluster, are thought to both be processed from a common pri-miR. Interestingly, three different species of pri-miR-221/222, approximately 2kb, 28kb and 108kb, have been described [51]. The expression of these three pri-miR-221/222 differs in different cell lines, however, publically available RNA-seq data from ENCODE revealed that in GM12878 (an EBV-immortalised LCL) the major pri-miR-221/222 to be expressed is the 28kb species (Fig 6A). Using LCLs described above (Figs 1 and 4) it was established that both EBNA3A and EBNA3C are necessary to up-regulate the 28kb pri-miR-221/222 (Fig 6B and 6C). The level of pri-miR-221/222 in those cells echoes the level of the mature miR-221/miR-222 detected (compare Figs 1 and 4 with Fig 6). Consistent with this, when 4HT was added to EBNA3A-ERT2 LCLs or EBNA3C-HT LCLs never HT, there was a significant up-regulation of the pri-miR-221/222 (Fig 6D and 6E). The activation of both EBNA3A and EBNA3C through addition of 4HT not only up-regulates the pri-miR-221/222, but also increases the expression of the mature miR-221 and miR-222 in these cells (S6 Fig). Furthermore, it was possible to show that EBNA3A and EBNA3C repress the well-characterised pri-miR-143/145 in the same LCLs (S7 Fig).

### ChIP-seq and ChIP-qPCR analysis of the miR-221/miR-222 and miR-143/miR-145 loci

**Fig 7:**
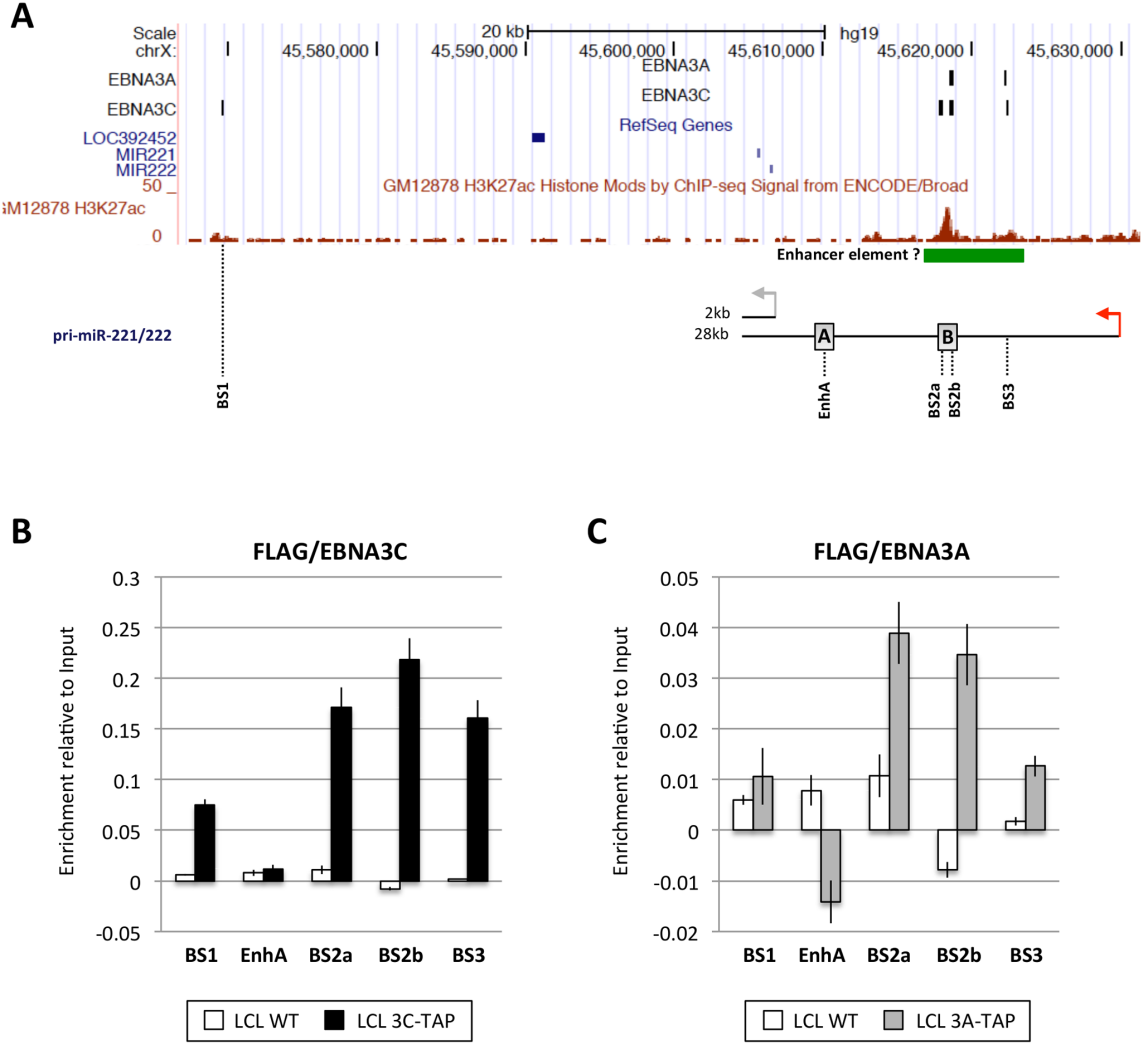
EBNA3A and EBNA3C bind near the miR-221/miR-222 locus. (**A**) ChIP-seq data at miR-221/miR-222 cluster genomic locus generated from LCL 3A-TAP and LCL 3C-TAP [27] were displayed using UCSC Genome Browser. EBNA3A and EBNA3C binding sites (BS1, BS2a, BS2b and BS3) are shown in black squares. The ChIP-seq data from ENCODE for H3K27ac in GM12878 is also displayed. Below it, a schematic representation of the 2kb and 28kb pri-miR-221/222 is shown. Grey and red arrows show the transcription start site of the 2kb and 28kb pri-miR-221/222 respectively. Positions of primer pairs used for qPCR to analyse precipitated DNA from ChIP are indicated, along with the positions of previously described enhancers (grey squares A and B) [47]. (**B**) ChIP qPCR analyses using anti-Flag antibody to precipitate 3C-TAP and chromatin associated with it in LCL 3C-TAP was performed. As a control for antibody specificity similar ChIP was performed using LCL infected with B95.8-BAC virus (LCL WT). No binding was detected at the enhancer A (EnhA) used as negative control. Values represent ratio of chromatin precipitated, after correction for IgG, relative to 2.5% of input. (**C**) As in (B) but using LCL 3A-TAP in order to precipitate 3A-TAP protein and associated chromatin. The ENCODE ChIP-seq data indicate a clear peak for RNA Pol II and TBP (https://genome.ucsc.edu; at coordinates ChrX: 45,629,468–45,629,838 GRCCh37/hg19) this is where the RNA-seq signal for pri-miR-221/222 (28kb) starts, so we have assumed this was the transcription start site (TSS) throughout our analysis.

EBNA3A and EBNA3C are viral transcription factors that can be targeted to host genes at sites proximal to transcription start sites (TSS) and/or distal regulatory elements and sometimes modulate looping of chromatin between these sites to modify gene expression [19,20,21,29]. Therefore, in order to determine whether the regulation of miR-221/miR-222 and/or miR-143/miR-145 might result from direct binding of either EBNA3A or EBNA3C –or both—to chromatin at the genomic locus of each miR cluster, genome-wide chromatin immunoprecipitation (ChIP) data sets were interrogated. ChIP-seq was performed using D11 LCLs expressing either an epitope-tagged EBNA3A (3A-TAP) or an epitope-tagged EBNA3C (3C-TAP) and the immunoprecipitation (IP) was performed using an anti-FLAG antibody; this was followed by high throughput DNA sequencing (S8 Fig and K. Paschos et al, manuscript in preparation; [27]). Analysis of the genomic locus including miR-221/miR-222 (chromosome Xq11.3) revealed a region located approximately 9kb downstream of the TSS for the 28kb pri-miR-221/222 that includes three binding sites for EBNA3C (sites BS2a, BS2b and BS3 in Fig 7A). One of these sites precisely overlapped an EBNA3A-binding site (BS2b) and one partially overlapped (BS3). Sites BS2a and BS2b are spaced only 1kb apart at a location previously reported to be a cis-acting enhancer element involved in the regulation of both miR-221 and miR-222 [47]. An additional EBNA3C-only binding site (BS1) was located about 60kb downstream of the pri-miR-221/222 TSS (Fig 7A). Robust binding of EBNA3C-TAP to sites BS1, BS2a, BS2b, and BS3 was confirmed by ChIP-qPCR (Fig 7B), but no binding was observed using control primers corresponding to another region previously described as an enhancer (EnhA [47], Fig 7A) Under similar ChIP-qPCR conditions, and again using the anti-FLAG antibody, significant binding of EBNA3A-TAP could also be detected in BS2a, BS2b and BS3, whereas no binding was detected for site BS1 or EnhA (Fig 7C). Taken together, these results identified an ‘intragenic’ region where both EBNA3A and EBNA3C bind to chromatin (BS2a, BS2b and BS3). ENCODE data (displayed on the UCSC genome browser) shows this region has high levels of the activation associated histone modification H3K27ac—providing further evidence that it probably acts as an enhancer of transcription in LCLs (Fig 7A).

In contrast, interrogation of ChIP-seq data corresponding to the miR-143/miR-145 locus (chromosome 5q.32)–over a region of more than 1Mb either side of the putative TSS of the pri-miR-143/145 –failed to reveal any EBNA3A- or EBNA3C-binding sites (compare S9 Fig with Fig 7A). Our interpretation of these data is that transcriptional regulation of pri-miR-143/145 (and hence mature miR-143/miR-145) is unlikely to be due to EBNA3A/EBNA3C binding to cis-regulatory elements and is therefore probably a secondary, trans-acting effect of the regulation of an unknown gene(s). However, we cannot rule out binding to extremely long-range regulatory elements.

### EBNA3A and EBNA3C increase the level of active chromatin markers around pri-miR-221/222 TSS

**Fig 8:**
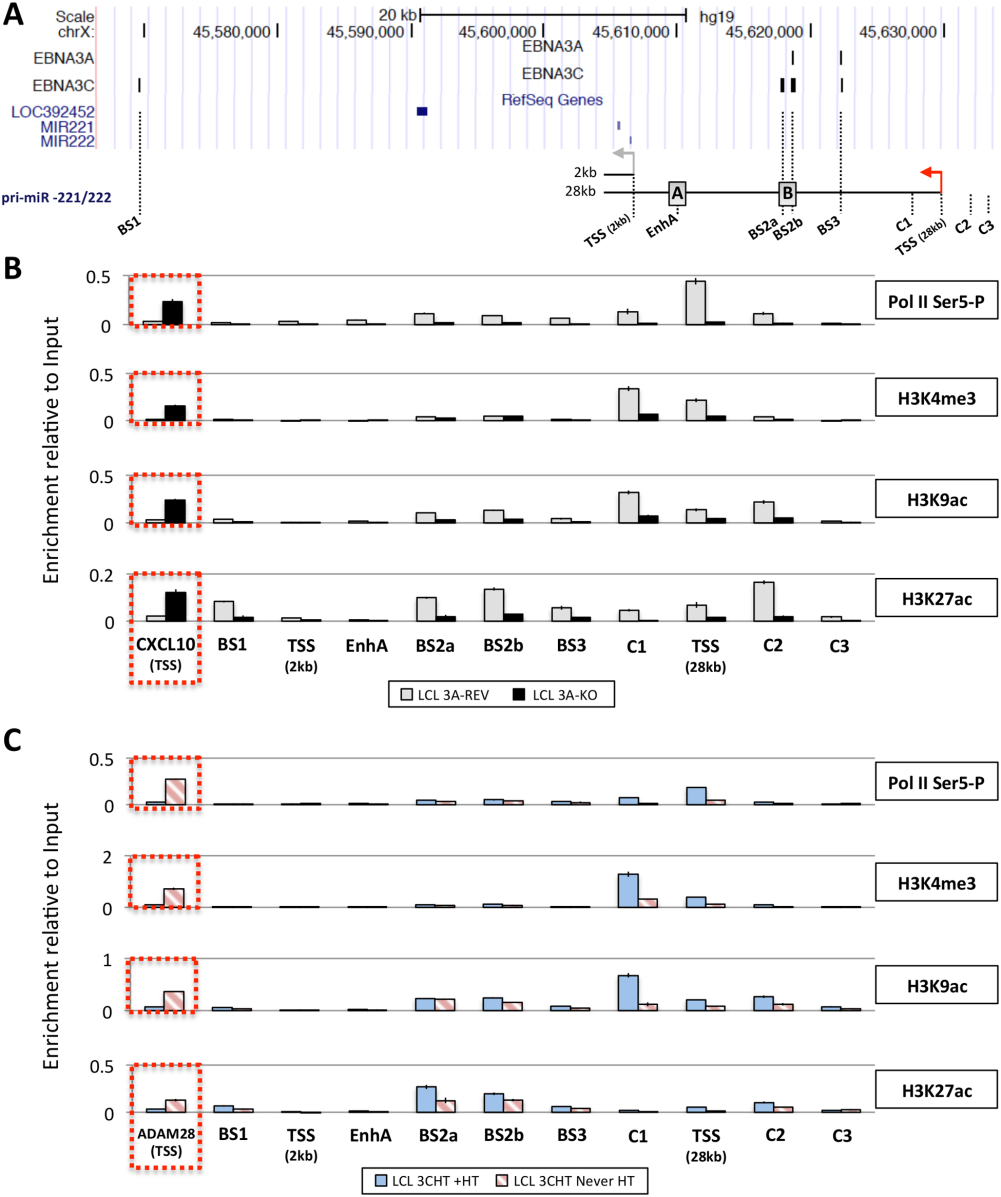
Active chromatin markers and RNA polymerase (Pol) II occupancy on the miR-221/miR-222 cluster genomic locus. (**A**) ChIP-seq data at miR-221/miR-222 cluster genomic locus generated from LCL 3A-TAP and LCL 3C-TAP were displayed using UCSC Genome Browser as in Fig 7A. A schematic representation of the 2kb and 28kb pri-miR-221/222 is shown with grey and red arrows representing each transcription start site (TSS). Positions of primer pairs used for qPCR to analyse precipitated DNA from ChIP are indicated along with the position of previously described enhancers (grey squares A and B) [47]. (**B**) ChIP was performed on extracts from EBNA3A-KO and EBNA3A-REV LCL (D3) and antibodies specific for phospho-Ser5 Pol II, H3K4me3, H3K9Ac and H3K27Ac were used. As a control for antibodies and cell lines, a primer pair for CXCL10 TSS was used. Values represent ratio of chromatin precipitated, after correction for IgG, relative to 2.5% of input. (**C**) As in (B) but using p16-null LCL 3CHT (LCL 3CHT never HT) cultured for 30 days with (LCL 3CHT +HT) or without 4HT. For the ChIP using LCL 3CHT, ADAM28 TSS primer pair was used as control.

Next, in order to determine whether the up-regulation of pri-miR-221/222 by EBNA3A and EBNA3C correlates with histone modification and chromatin remodeling, ChIP analyses were performed on EBNA3A-KO and EBNA3A-REV LCLs as well as EBNA3C-HT LCLs (never HT) or treated with 4HT (Fig 8). Initially the phosphorylation of the RNA polymerase II at serine-5 (Ser5)–that indicates transcriptional initiation and activation [77]–was investigated (Fig 8B and 8C). This revealed that the level of phospho-Ser5 Pol II is elevated around the TSS of 28kb pri-miR-221/222 only when both EBNA3A and EBNA3C are expressed and functional (in EBNA3A-REV and EBNA3C-HT cultured with 4HT). No binding was seen at the putative 2kb TSS. Primers that amplify CXCL10 TSS and ADAM28 TSS were used as controls for EBNA3A and EBNA3C repressed genes respectively and found, as expected, a higher level of phospho-Ser5 Pol II only when EBNA3A or EBNA3C were absent/non-functional. We then performed ChIP analysis for marks of active chromatin (H3K4me3, H3K9ac and H3K27ac) across the miR-221/miR-222 cluster locus (Fig 8B and 8C). As expected, a higher level of activation marks was found around the 28kb pri-miR-221/222 TSS only when functional EBNA3A and EBNA3C were expressed. Again there were no changes around the putative 2kb TSS that suggest it is regulated. Interestingly, histone activation marks where higher (in particular H3K9ac and H3K27ac) at BS2a and BS2b sites, that is the region previously described as an enhancer for miR-221/miR-222, only when both EBV proteins are functional. Taken together these data are consistent with increased miR-221/miR-222 expression occurring when EBNA3A and EBNA3C are expressed in an active form, bind chromatin at specific sites and alter the epigenetic profile of the locus.

### EBNA3A and EBNA3C induce expression of the 28kb pri-miR-221/222 by promoting the formation of enhancer-promoter looping

**Fig 9:**
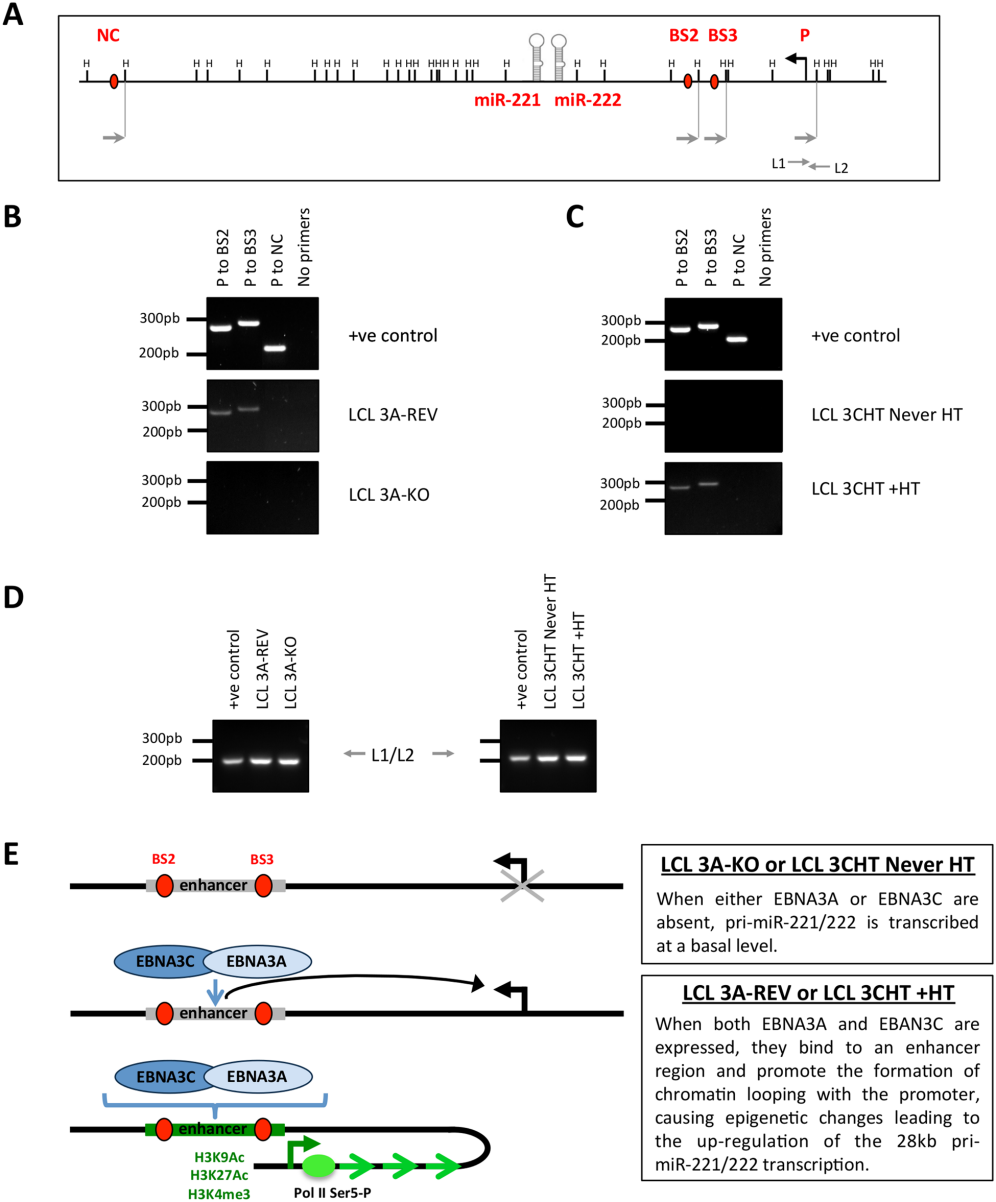
EBNA3A and EBNA3C induce chromosome looping at the miR-221/miR-222 cluster locus. (**A**) Schematic of the miR-221/miR-222 cluster locus depicts the location of both miRs, the HindIII sites, the 28kb pri-miR-221/222 transcription start site and primers used for the chromosome conformation capture assay. (**B**) EBNA3A-KO and EBNA3A-Rev LCLs (D3) were used for chromosome conformation analysis. Interaction between the promoter (P) of the 28kb pri-miR-221/222 and EBNA3A/3C binding site 2 (BS2 –including BS2a and BS2b, see Fig 8A) or 3 (BS3) is dependent on the presence of EBNA3A. PCR primers (NC) corresponding to a site located downstream of the miR-221/miR-222 locus were used as a negative control. Positive control (+ve control) showed PCR reactions using a DNA control template. (**C**) Same as (B) but using p16-null LCL 3CHT (LCL 3CHT never HT) cultured for 30 days with (LCL 3CHT +HT) or without 4HT. Interaction between P to BS2 and P to BS3 occurred only when EBNA3C is active (LCL 3CHT +HT). (**D**). Loading control primers L1 and L2 amplify DNA contained in a single HindIII fragment and have been used as DNA loading control between the DNA samples used for chromosome conformation capture. (**E**) Schematic model of chromatin loop formation induced by EBNA3A and EBNA3C at miR-221/miR-222 cluster locus.

It has recently been shown that the EBNA3 viral proteins can regulate transcription by modulating enhancer-promoter loop formation [20]. So in order to determine whether the EBNA3s could either promote or disrupt the formation of looping between ‘intragenic’ enhancer elements (BS2 and BS3 –where both EBNA3A and EBNA3C bind) and the promoter of the 28kb pri-miR-221/222, chromosome conformation capture (CCC) analysis was performed. A schematic map of the miR-221/miR-222 genomic locus with the location of the HindIII restriction sites and PCR primers is shown in Fig 9A. The CCC results showed looping interactions between regions BS2 and BS3 and the promoter, only in EBNA3A-REV LCL and EBNA3C-HT LCL treated with 4HT (Fig 9B and 9C); that is in cells in which both EBNA3A and EBNA3C are active and the 28kb pri-miR-221/222 is up-regulated. No looping was found between a control region (NC) and the promoter for 28kb pri-miR-221/222, but a control PCR product L1/L2 was found in all samples showing that equal amounts of DNA were present in all reactions (Fig 9D). These results demonstrate that EBNA3A and EBNA3C are both required for the formation of looping between two sites within an enhancer region and the 28kb pri-miR-221/222 promoter, leading to increased transcription of the pri-miR (Fig 9E).

### miR-221/miR-222 inhibition of p57^KIP2^ expression in LCLs requires EBNA3A and EBNA3C

MiR-221 and miR-222 have been described as oncogenic miRs (oncomirs) because they are often expressed at high levels in cancer (see Introduction). Furthermore it has been demonstrated in various types of non-B cell that they can target mRNAs corresponding to several tumour suppressor genes and promote their translational inhibition and/or degradation. There are multiple reports that the CDKIs p57^KIP2^ and p27^KIP1^ are targets and a single report of the pro-apoptotic p53-response protein PUMA in epithelial cells [50,56,57,58,59,76].

**Fig 10:**
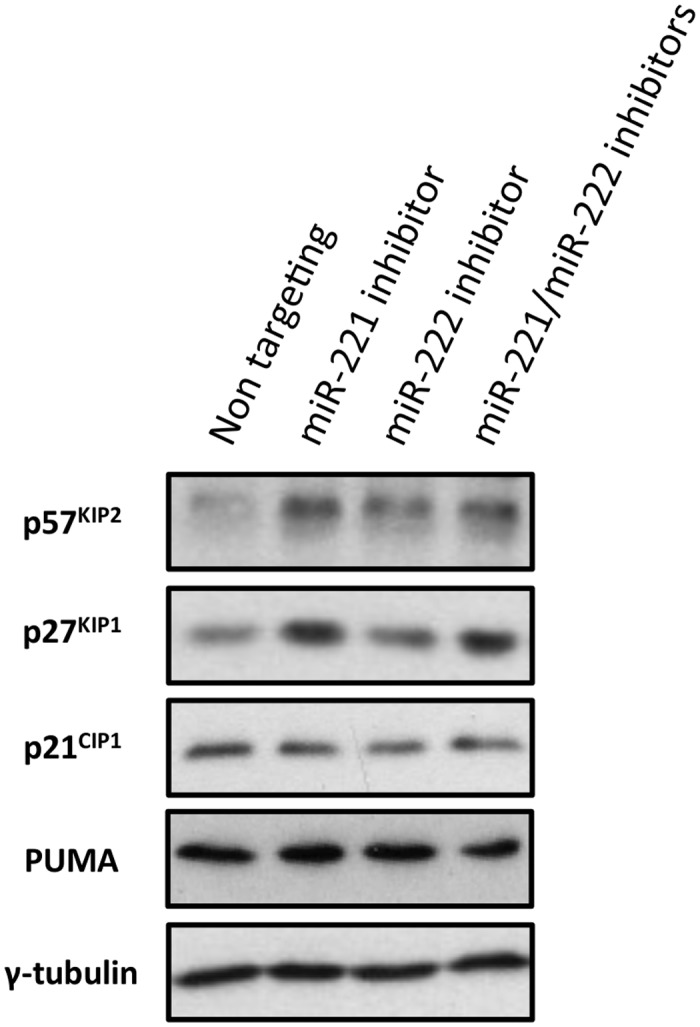
Inactivation of miR-221 and miR-222 in LCLs with corresponding anti-miRs. LCLs were electroporated with the anti-miR indicated; p57^KIP2^, p27^KIP1^ p21^CIP1^ and PUMA expression have been analysed by western blot. The blot was probed for γ-tubulin as an additional control for loading and a non-targeted protein. There were increases in 57^KIP2^ and p27^KIP1^, but not p21^CIP1^ or PUMA in cells transfected with the specific LNA anti-miRs.

We first determined whether p57^KIP2^ and p27^KIP1^ were miR-221/miR-222 targets in LCLs. To do this EBNA3A-REV cells were electroporated with LNA anti-miR-221, anti-miR-222, both anti-miRs, or a negative control. The electroporation of anti-miR-221, anti-miR-222 or both was accompanied by an increase in p57^KIP2^ protein level, and p27^KIP1^ increased only when miR-221 was inhibited (Fig 10). For comparison we also analysed expression of the related CDKI, p21^CIP1^ (not known to be a miR-221/miR-222 target) and found no change when the miRs were inhibited. The level of PUMA was also unaltered in this B cell context. These depletion experiments relied upon poor transfection efficiencies common to all LCLs, therefore in most cells of the population the specific miRs were not inactivated. Nevertheless taken together, the data established to our satisfaction that p57^KIP2^ and (to a lesser extent) p27^KIP1^ are targets of the miR-221/miR-222 cluster in EBV-transformed human B cells.

**Fig 11:**
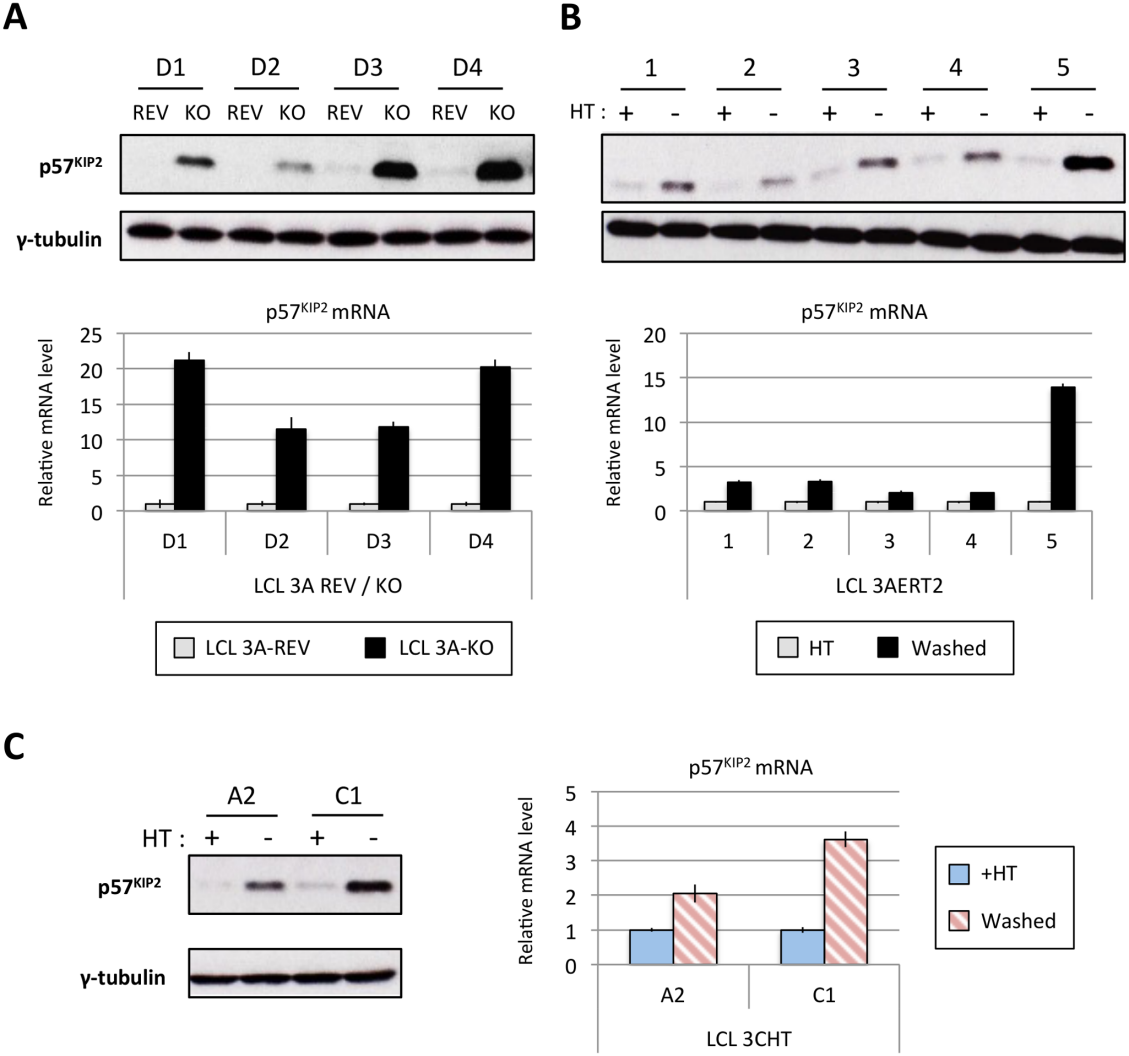
EBNA3A and EBNA3C regulate p57^KIP2^ expression. (**A**) Western blot showing the level of p57^KIP2^ expression in four EBNA3A-KO (KO) LCLs and their revertant equivalent (REV) (used in Fig 1). Below each Western blot is shown the level of p57^KIP2^ mRNA determined by qPCR, normalized to GN2BL1 and relative to each “wild type” cell LCL EBNA3A-REV (3A-REV) of which the mRNA level is set to 1. (**B**) As in (A) but using five EBNA3A-ERT2 conditional LCLs cultured with (+) or without (-) 4HT (used in Fig 4). (**C**) Western blot showing the expression of p57^KIP2^ protein (left panel) and qPCR showing the level of p57^KIP2^ mRNA (right panel) in two p16-null LCL 3CHT cultured with (+) or without (-) 4HT (used in Fig 1).

The expression of p57^KIP2^ was therefore subjected to more detailed and stringent analyses. The results, (compiled in Fig 11), unambiguously demonstrated across multiple LCLs carrying either EBNA3A-KO or-revertant EBV, or LCLs conditional for EBNA3A or EBNA3C, that p57^KIP2^ protein expression is almost completely ablated when EBNA3A and EBNA3C are both active. However, if either of these EBNA proteins is absent or inactivated, substantial amounts of p57^KIP2^ can be detected. Analysis by qPCR also showed significant increases of mRNA corresponding to p57^KIP2^ in the absence of functional EBNA3A or EBNA3C, but the degree of regulation was rather more variable between cell lines than was the protein expression (Fig 11). These results suggest that miR-221 and miR-222 not only block translation, but might also enhance the degradation of p57^KIP2^ mRNA (both mechanisms of action have been described [33,34].

MiR-221 and miR-222 have also been reported to regulate p27^KIP1^ in non-B cells. In our miR inhibition assay (Fig 10) we showed that p27^KIP1^ increased after miR-221 inhibition but not miR-222. Although we see regulation of p27^KIP1^ protein levels by EBNA3A in LCLs, it is neither as robust nor quite as consistent as the regulation of p57^KIP2^ (for examples see S10 Fig)—currently we do not know the reasons why p27^KIP1^ and p57^KIP2^ are differentially regulated in these lines.

### Induction of p57^KIP2^ coincides with reduced phosphorylation of the Rb protein and cell proliferation in EBNA3A-ERT2 cells minus 4HT

**Fig 12:**
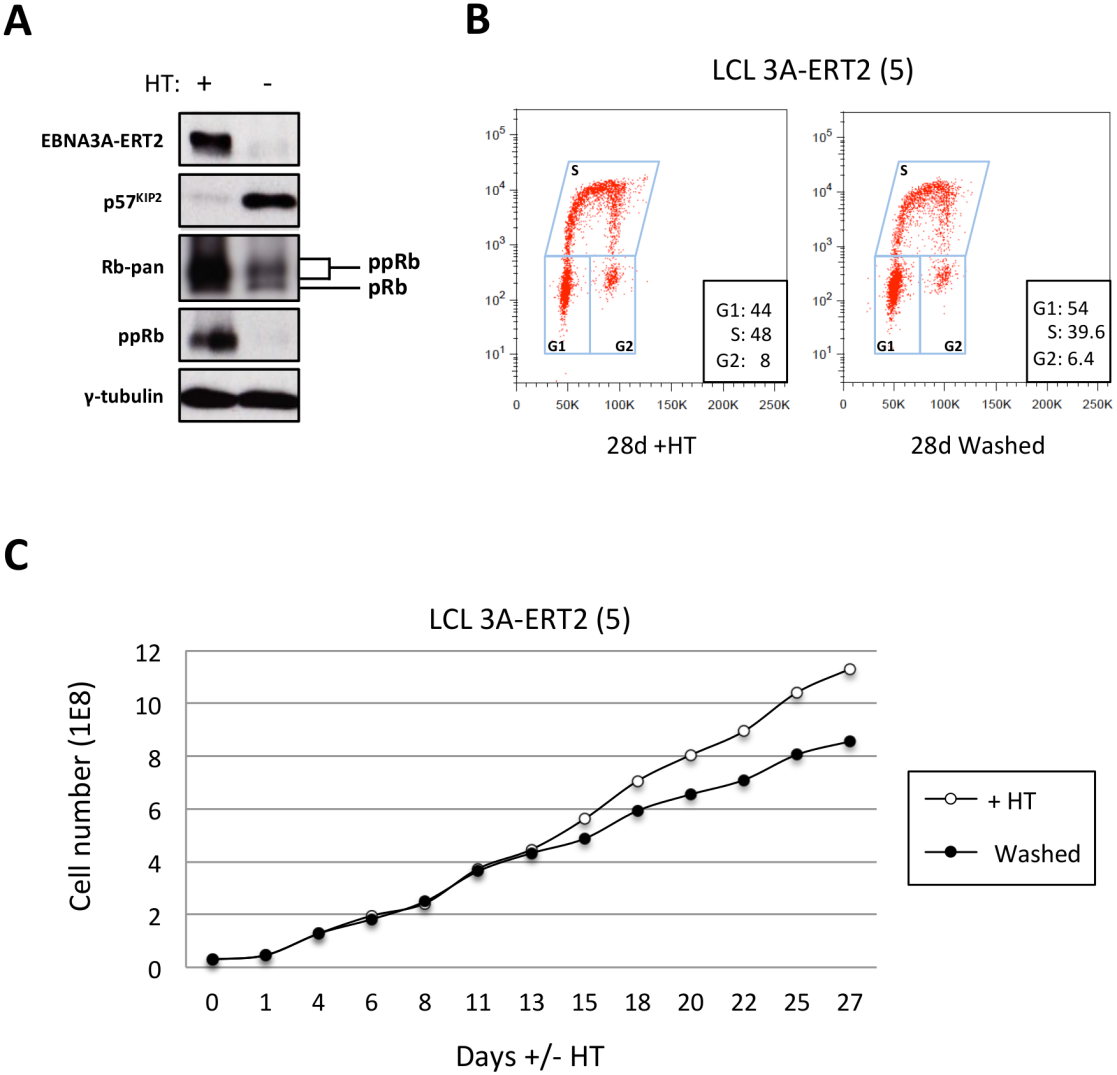
The increase of p57^KIP2^ level is associated with de-phosphorylation of Rb and reduced entry into S phase and proliferation. (**A**) Western blot analysis of extracts from LCL EBNA3A-ERT2 (line 5) cultured with (+) or without (-) 4HT for 30 days showing that after inactivation (and degradation) of the EBNA3A-ERT2 fusion protein, p57^KIP2^ expression increases, and the amount of hyperphosphorylated Rb (ppRb) is dramatically reduced and is no longer detected using a phospho-Rb-specific antibody. The blot was probed for γ-tubulin as a control for loading. (**B**) Cell cycle distribution of LCL EBNA3A-ERT2 (line 5) culture with or without 4HT for four weeks was determined by flow cytometry following exposure to EdU for 2 hours (2h pulse). (**C**) A comparison of the population growth rate between these cells cultured with or without 4HT was analysed by counting the number of viable cells every 2–3 days. Total cell numbers were plotted at each time point. Data are representative of two independent experiments.

In order to determine the consequences of up-regulating p57^KIP2^ in LCL cells that have grown out after infection of normal primary B cells with the minimum of selection, an early passage (<2 months post-infection) EBNA3A-ERT2 line produced from a mixed donor population of B cells (LCL 5) was used. This line was established in the presence of 4HT and the cells express little or no p57^KIP2^, but on removal of 4HT from the culture medium, they soon produce substantial amounts of p57^KIP2^ protein (Figs 11B and 12A). Consistent with the increase in p57^KIP2^ there was a pronounced reduction in the phosphorylation of the tumour suppressor Rb and this was associated with a gradual reduction in proliferation as revealed by reduced DNA synthesis (EdU incorporation) and reduced cell population growth (Fig 12). Similar analysis was repeated on several other independent EBNA3A-conditional LCLs and produced essentially identical results (S11 Fig). An important enzyme necessary for the phosphorylation of Rb is the cyclin-dependent kinase CDK6. Therefore the most likely explanation for the inhibition of Rb phosphorylation is the binding of p57^KIP2^ to CDK6 resulting in inactivation of the latter (see co-immunoprecipitations in S12 Fig). The effect of low levels of miR-221/miR-222 and high levels of p57^KIP2^ on the proliferation of these cells was surprisingly modest, rather less than has been reported previously in EBNA3A-conditional LCLs when EBNA3A was inactivated [10,78]; we do not know the reason for this, but as we have indicated above, different lines can have different properties because of clonal variation. The roles and interactions of the various CDKIs regulated by EBV are discussed in more detail below.

## Discussion

Using a reverse genetics approach, made possible by the use of LCLs carrying knockout, revertant or conditional-EBV recombinants, we have explored the roles of the EBNA3 proteins in the regulation of cell miRs in B cells. This has revealed that both EBNA3A and EBNA3C –but not EBNA3B –are required for the transactivation of the oncomiRs miR-221 and miR-222, while concurrently silencing the expression of the tumour suppressor miR-143/miR-145 cluster.

Both EBNA3A and EBNA3C were shown by ChIP experiments to associate with multiple sites in a genomic region about 19kb upstream of the miR-221/miR-222 coding sequences and about 9kb downstream from the pri-miR TSS that is dominant in LCLs. These EBNA3 binding sites correspond to a region previously identified functionally and by histone modifications as an enhancer of transcription. So the data are consistent with the miRs being directly transactivated by the combined action of EBNA3A and EBNA3C. This cooperation between these two EBNA3 proteins to modulate transcription is reminiscent of the regulation of many host protein-encoding genes in EBV-infected LCLs [14,20,28,29]. At this stage it is not possible to say what factors are responsible for recruiting EBNA3A and or EBNA3C to BS2a, BS2b and BS3, nevertheless ENCODE data indicate >20 transcription factors that could bind at these sites in the GM12878 LCL (S13 Fig). This list includes many factors previously reported to be involved in the recruitment of EBNAs to sites across the human genome (eg ATF2, BATF, PAX5, RUNX3 and SPI1 [19,20,29]).

Since EBNA3A and EBNA3C were found to bind to multiple sites in a previously characterised enhancer element—we assumed, and then confirmed, that transactivation involves modulation of the local three-dimensional architecture of chromatin (long-range ‘looping’) that brings the enhancer elements into contact with the TSS of the 28kb pri-miR only when functional EBNA3A and EBNA3C are present (see Fig 9). Similar topological changes have been reported in the regulation of protein encoding genes such as the *ADAM28/ADAMDEC1* locus [20,21]. However, at most of the EBNA3A/EBNA3C regulated genes that have been well characterised [for example BIM (*BCL2L11*), p16^INK4a^ (*CDKN2A*) and the *ADAM28/ADAMDEC1* locus] transcription is repressed; and this is probably because these two viral proteins can recruit cellular co-repressors such as HDACs, CtBP and components of polycomb protein complexes (see Introduction). On some genes they can also displace the EBV transactivator EBNA2, resulting in substantially reduced transcription [20,21]. Here, for the first time, we describe a long-range enhancer—promoter interaction mediated by EBNA3A and EBNA3C resulting in increased transcription, ie activation. But very little is known about how EBNA3A and EBNA3C together might activate transcription, although it is probably related to the their capacity to physically interact with each other [27] and perhaps recruit co-activators such as the histone acetyltransferase p300 [22]. This mechanism would be consistent with the increase in acetylation seen on histone H3 lysine-27 (H3K27ac) at the enhancer binding sites and around the promoter when the miR-221/miR-222 locus is activated (Figs 7A and 8). The consensus of opinion is that in this type of gene regulation—that involves chromatin looping—cellular repressors or activators are recruited in a context-specific manner, but what cofactors, features of genomic sequence and chromatin topology determine whether the outcome is repression or activation, remain largely unknown (reviewed in [79]).

It has been previously reported that EBV can induce expression of miR-221/miR-222 [80,81] and that the latency-associated protein LMP1 can activate miR-221/miR-222 expression after single gene transfer into BL-derived cells [82]. Moreover, the cluster can also be activated by NF-kB [47]. Since LMP1 is expressed in all the LCLs used in this study (see for example Fig 3) and activates NF-kB signaling, we cannot rule out the possibility that signal transduction from this viral membrane protein also contributes to the activation of the 28kb pri-miR. It is possible that EBNA3A and EBNA3C acting together play a role in reorganizing local chromatin in order to potentiate LMP1 and NF-kB-mediated transactivation of miR-221/miR-222.

Silencing of the miR-143/miR-145 locus by the combined action of EBNA3A and EBNA3C remains poorly understood. Since no EBNA3 binding sites were detected within more than a million DNA base pairs either side of the pri-miR-143/145 TSS (S9 Fig), it is likely that this repression of transcription is a secondary, downstream event triggered by altered expression of another EBNA3A/EBNA3C-target gene. However, we are currently unable to formally test this.

The two miR clusters described here are deregulated in multiple human cancers, including B cell lymphomas (see Introduction) and therefore probably have the potential to influence B cell proliferation, transformation, and EBV-associated lymphomagenesis. The details of how the tumour suppressors miR-143 and miR-145 might inhibit cell proliferation are poorly understood. They co-operatively promote differentiation and repress proliferation in several cancer and primary cell lines and are both up-regulated during senescence in human fibroblasts [60,61,62]. It may be significant that miR-143 and miR-145 are also repressed by the E7 oncoprotein in epithelial cells infected with human papillomavirus (HPV)-31 [83]. Although various target mRNAs have been proposed, there appears to be a lot of cell-type specificity and a general lack of consensus on precisely how miR-143/miR-145 act as tumour suppressors. There are very few data available on the activities of miR-143/miR-145 in B cells, although there has been one report of their down-regulation in EBV-transformed, but not in mitogen-stimulated B cells [84].

**Fig 13:**
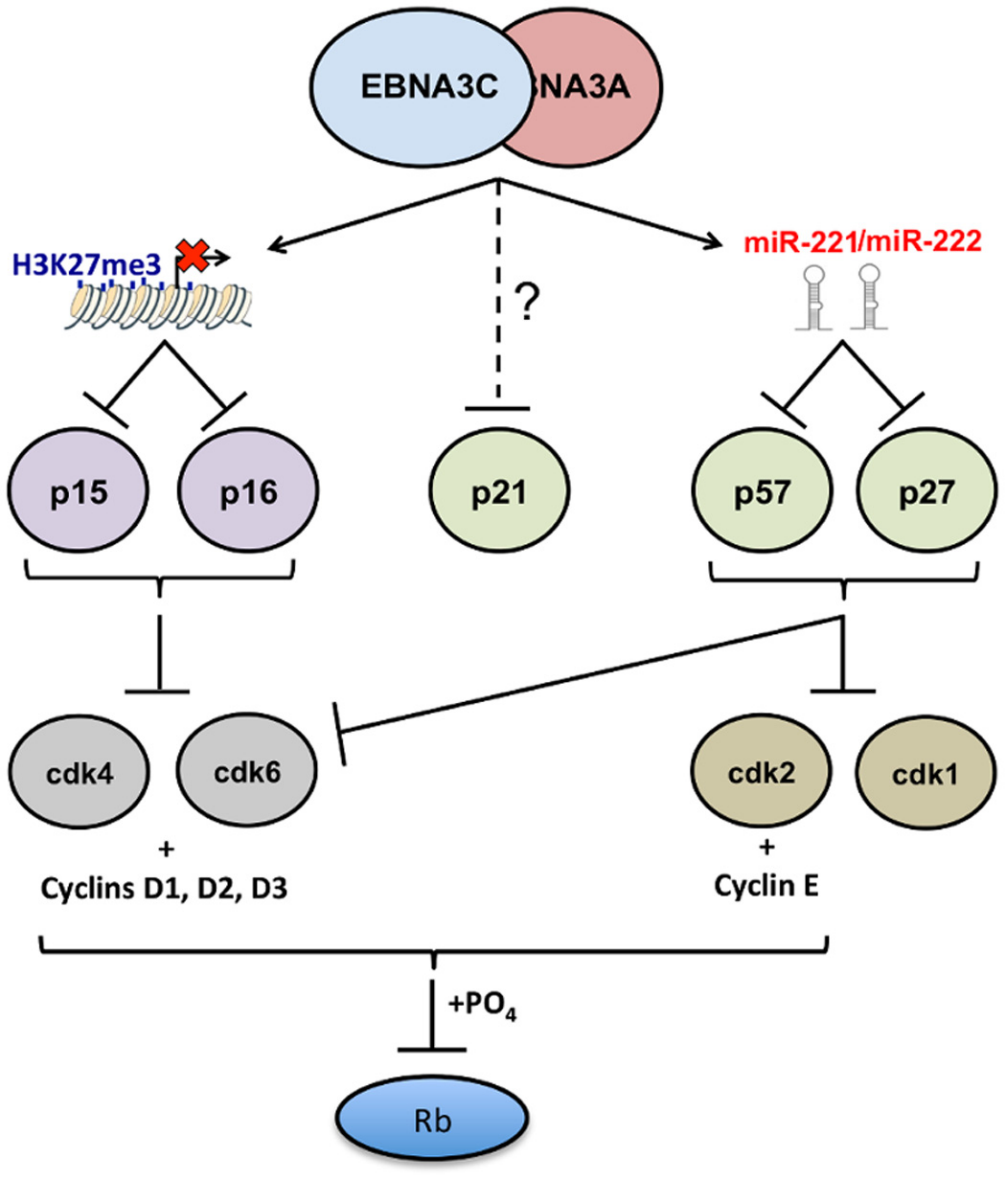
EBNA3A and EBNA3C together modulate the activity of multiple cell cycle inhibitors by more than one mechanism. Schematic representation of how, by acting together, EBNA3A and EBNA3C regulate members of two families of cyclin-dependent kinase inhibitors (CDKIs). EBNA3A and EBNA3C jointly repress expression of the two closely related INK4 CDKIs p16^INK4a^ (encoded by *CDKN2A*) and p15^INK4b^ (encoded by *CDKN2B*). In both cases repression of transcription is associated with the tethering of EBNA3A and EBNA3C to chromatin in the region of the *CDKN2A/CDKN2B* locus [19,30,32]. Inhibition of transcription involves the polycomb-mediated epigenetic histone modification H3K27me3. In this report the same combination of EBNA3 proteins has been shown to repress two members of the second (CIP/KIP) family of CDKIs. Here repression of protein expression is not directed at the transcription of the genes for p57^KIP2^ (*CDKN1C*) and p27^KIP1^ (*CDKN1B*), but is post-transcriptional and mediated by the miR-221/miR-222 cluster that is directly transactivated by EBNA3A and EBNA3C. The biochemically related proteins p16^INK4a^ and p15^INK4b^ both have the capacity to inhibit CDK4 and CDK6 activity. The CIP/KIP kinases are more promiscuous and target, in addition, other CDKs including CDK2. Recently there have been reports that EBNA3A and EBNA3C can each also regulate the third member of the CIP/KIP family, namely p21^CIP1^ [85,86] but the mechanisms in LCLs have not been clearly defined.

In contrast to miR-143/miR-145, functions of the miR-221/miR-222 cluster is relatively well characterised, with wide agreement that two of the major targets are mRNAs for CIP/KIP CDKIs p57^KIP2^ and p27^KIP1^, the translation of which are robustly inhibited by these miRs in various types of cell (see Introduction). We, and others, had previously shown that EBNA3A and EBNA3C can block transcription of two members of the INK4 CDKI family, p16^INK4a^ and p15^INK4b^ [12,30,32,78]. Furthermore it has been reported that these same two EBV proteins might also repress expression of a third member of the CIP/KIP family, p21^CIP1^ [85,86]. Since intrinsic cell cycle inhibitors are emerging as important targets of EBNA3A and EBNA3C, we focused on the consequences of miR-221/miR-222 induction in LCLs. Utilizing multiple cell lines carrying either knockout mutant viruses or viruses conditional for EBNA3A or EBNA3C expression, we have clearly established that in B cells, transformed by and latently infected with EBV, both EBNA3A and EBNA3C are necessary to inhibit expression of p57^KIP2^ and (less robustly and reproducibly) p27^KIP1^. Both of these proteins can act as tumour suppressors by reducing cell proliferation because they target and inhibit CDKs 2,4,6 (see schematic in Fig 13; reviewed in [87]). Reducing the expression of these CIP/KIP CDKIs via miR-mediated inhibition is therefore likely to enhance the proliferation of LCLs and might play a role in establishing EBV persistence in B cells *in vivo*. It could also contribute to the development of EBV-associated B cell lymphomas such as those in the immunocompromised, in DLBCL [52,53,54] and in the sub-group of BL that express the EBNA3s (known as Wp-restricted BL [88]).

It should also be noted that in many of the LCLs used in this study—particularly those passaged for prolonged periods—induction of p57^KIP2^ had little or no effect on proliferation. We suspect in some cases this is because genetic or epigenetic impairment of the Rb tumour suppressor hub is readily selected in the expansion of these rapidly proliferating cell populations (see Introduction). Alternatively, p57^KIP2^ may not always have a significant anti-proliferative effect in activated B cells and may sometimes produce more subtle phenotypes. It is important to remember that EBNA3A and EBNA3C together repress multiple cell cycle inhibitory pathways that culminate in the inactivation—by phosphorylation—of the tumour suppressor protein Rb (Fig 13); that is, there appears to be considerable redundancy. The currently available data suggest that p16^INK4a^ is the major CDKI to be targeted in B cell transformation [30,31], but it now appears that p15^INK4b^, p57^KIP2^, p27^KIP1^ and p21^CIP1^ might all have to be controlled in the molecular balance required for the establishment of EBV latency. It will require painstaking analysis using EBV with specific mutations in EBNA3A and EBNA3C and shRNA and/or gene-editing technologies to dissect and resolve these effects on B cell proliferation, senescence and perhaps differentiation.

In summary, it appears that during the co-evolution of EBV and its host, two cooperating factors (EBNA3A and EBNA3C) have emerged to control transcription of not only host cell genes, but also long non-coding pri-miRs and miRs. It is remarkable that two distinct families of cell cycle inhibitory factors—the INK4 and CIP/KIP CDKIs—are a specific focus in this double-routed regulation of the host proteome. The implication is that extending the proliferation of B cells via the activities of EBNA3A and EBNA3C is a very important feature of EBV biology *in vitro* and therefore *in vivo*.

## Materials and Methods

### MicroRNA profiling by TaqMan quantitative real-time PCR low density array (TLDA)

RNA was isolated using *mir*Vana miRNA isolation Kit (Ambion) including the optional step for enrichment of small RNA (<200bp), which enables more sensitive detection of low-level small RNAs. Taqman MicroRNA RT kit and Megaplex Primer Pool A (ABI) were used to reverse transcribe up to 381 microRNAs in a single reaction according to the manufacturer’s instructions. Generally 300ng of RNA was reverse transcribed per reaction and the cDNA product was used in qPCR without pre-amplification.

Three hundred and seventy seven human microRNAs were profiled by real-time qPCR using the Taqman MicroRNA A Card v 2.0 (ABI). cDNA was diluted, mixed with Taqman Universal PCR Master Mix II (ABI) and loaded into the pre-configured micro-fluidic card. Real-time reaction was run on a 7900HT Real-Time PCR System (ABI) and data analyzed using the SDS RQ manager software (ABI).

### Construction of recombinant EBV-BAC EBNA3A-ERT2

An EBNA3A-ERT2 fusion protein (3A-ERT2) was constructed in the B95-8 EBV background using the 4-hydroxytamoxifen-sensitive human estrogen receptor ERT2 containing the G400V/M543A/L544A triple mutation [89]. The connection between EBNA3A and ERT2 is a small linking sequence of 9 amino acids (GTGGVGQD) between the last amino acid of EBNA3A and amino acid 281 of ERT2. This fusion was recombined into the B95-8 bacterial artificial chromosome (BAC) using previously described methods [12,90,91] to produce BAC containing 3A-ERT2.

### Cell culture

Established LCLs were cultured in RPMI-1640 medium (Invitrogen) supplemented with 10% fetal calf serum, penicillin and streptomycin. LCL 3A-ERT2 and 3CHT were cultured with addition of 400nM of 4-hydroxytamoxifen (4HT, Sigma) where stated. After the infection of primary B cells, LCLs were grown to a volume and density suitable for freezing multiple aliquots (typically about 60ml at a density of 3×10^5^ cells/ml or greater). This took 4–6 weeks for 3A-ERT2, 3CHT, revertant LCLs and 6–12 weeks for the 3A-ERT2 grown without 4HT and EBNA3A mutant LCLs. LCLs 3CHT established in a p16-null background are described in [30]. Cells recovered from liquid nitrogen were cultured for at least 10 days (with 4HT if necessary) before the start of any experiment. At the end of an experiment the cells were discarded. Twenty-four hours before any experimental treatment, cells were seeded at a density of 3×10^5^ cells/ml.

### Infection of 1°B cells with recombinant EBV

Recombinant viruses were constructed and produced as described previously [12,91]. Primary B cells for the generation of LCL 3A-ERT2 and EBNA3-knockout and revertant LCLs were isolated from anonymous buffy coat residues (UK Blood Transfusion Service) by centrifugation over Ficoll. EBNA3A-knockout and revertant LCLs were made by infection of CD19+ B cells from four independent donors (D1, D2, D3, D4). LCL 3A-ERT2 line 5 was made by infection of CD19+ B cells from mixed donors. D11 (lines 1 and 2) and D13 (lines 3 and 4) 3A-ERT2 LCLs were made by infection of PBLs isolated from donors D11 and D13 with EBNA3A-ERT2 virus. To produce all LCLs, between 50μl and 1ml of virus was added to 10^6^ PBLs or 3×10^6^ CD19+ purified B cells in a well of a 24 well plate, and cultured initially in RPMI, supplemented with 15% FCS and with Cyclosporine A (500 ng/ml) for the first 2 weeks. Once LCLs had grown out into large culture volumes, the FCS level in the medium was reduced to 10%.

### Flow cytometry

Cell proliferation analyses were performed as described previously [30] by measuring the incorporation into DNA of nucleotide analogue EdU during a 2h pulse (Life Technologies). Cell fluorescence was measured on LSR II (Becton Dickinson) flow cytometer. Single cells were gated based on FxCycle Far Red fluorescence—Life Technologies (comparing fluorescence area to width or height at 633/690). The LIVE/DEAD Fixable Violet stain (Life Technologies) was used to determine the viability of cells (Fluorescence measured by 405/450 filters indicated live/dead status), and only live cells were included for the assessment of proliferation by EdU (488/530).

### Western immunoblotting

SDS polyacrylamide gel electrophoresis and Western blotting was performed essentially as described previously [12,27,91]. In some cases the membrane used for Western blotting was cut horizontally after protein transfer in order to facilitate multiple antibody probes and a single loading control for each blot.

### Co-immunoprecipitation assays

LCLs were harvested and lysed in immunoprecipitation (IP) buffer (50mM Tris-HCl pH 7.5, 150mM NaCl, 1mM DTT and 0.5% Nonidet P-40) plus protease inhibitors (Roche Molecular Biochemicals). Protein concentration was estimated colorimetrically using the Bio-Rad detergent-compatible assay and 250μg were used per IP. Cell extracts were then pre-cleared with 30μl of Protein G-Sepharose beads (GE Healthcare) at 4°C for 1h. Complexes were precipitated with specific antibodies (S1 Table) and the mixture was incubated at 4°C overnight. Then, 30μl of protein G-Sepharose beads were added for 1h at 4°C, washed four times in IP buffer and the immunopurified proteins were resolved by SDS-PAGE and detected by western blot.

### MicroRNA, mRNA and quantitative real time PCR (qPCR)

For qPCR, total RNA (including microRNA) was extracted from approximately 5×10^6^ cells for each cell line using the miRNeasy mini kit from Qiagen and following the manufacturer's instructions. Expression of miR-221, miR-222, miR-143, miR-145 and two snRNAs, RNU6B and RNU48 were quantified by qPCR using the TaqMan MicroRNA Assay listed in S2 Table (Applied Biosystem). Briefly, cDNA was synthesised from 10ng of total RNA using Taqman miRNA primers and the TaqMan MicroRNA Reverse Transcription Kit. qPCR was then performed using the TaqMan Universal PCR Master Mix. The cycling conditions were 95°C for 10min, followed by 45 cycles of 15sec at 95°C and 60sec at 60°C.

For mRNA analysis, one microgram of each RNA sample was reverse-transcribed using SuperScript III First-Strand Synthesis Supermix for qPCR (Invitrogen). 10 ng of cDNA product was then used per qPCR reaction (except for the detection of pri-miR-143/145 where 100ng were used) using Platinum Sybr Green qPCR SuperMix UDG kit (Invitrogen). Dissociation curve analysis was performed during each run to confirm absence of non-specific products. Sequences of the primers used are listed in S3 Table.

All qPCR were performed on an ABI 7900HT real-time PCR machine. GNB2L1 and RNU6B were used as endogenous controls for mRNA and miR respectively. Relative mRNA or miR expression was calculated using the comparative Ct (ΔΔ*C*T) method. The calculated errors in the graphs are the standard errors from three replicate qPCR reactions for each mRNA or miR.

### Chromatin immunoprecipitations (ChIP)

ChIP assay and qPCR analysis were performed essentially as described previously [27]. Antibodies and sequences of the primers used in these assays are listed in S1 and S4 Tables respectively.

### Chromosome conformation capture assay

Chromosome conformation capture assay was performed as previously described [92,93] with minor modification. Briefly, ten millions LCL were filtered through a 70μm cell strainer to obtain a single-cell preparation and fixed in 1% formaldehyde for 30 minutes at room temperature. The fixation reaction was stopped by quenching with 0.125M glycine, cells were washed twice with cold PBS containing protease inhibitors, re-suspended in 500μL of lysis buffer and lysed for 10 minutes on ice. Nuclei were collected and digested with 400 units of HindIII (20,000 U/mL, New England Biolabs) overnight at 37°C. The restriction digest reaction was stopped by addition of SDS (1.6% final concentration) and incubation at 65° for 30 minutes. The intramolecular ligation was performed by adding 100 Weiss units of T4 DNA ligase to the 10-fold diluted sample for 4h at 16°C followed by a 45 minutes incubation at room temperature. Protein digestion and reverse cross-linking was performed with overnight incubation at 65°C with 300μg proteinase K. RNA was then degraded with 300μg RNase for 1h at 37°C. Finally the DNA was twice phenol/chloroform extracted and ethanol-precipitated. Purified DNA was analysed by conventional PCR.

The generation of control template for ligation products was performed as previously described [20]. In brief, DNA regions covering the restriction sites of interest were PCR amplified, purified, mixed in equimolar amount and subjected to digestion with HindIII for 2 hours. The digested PCR products were ligated with 10 Weiss unit of T4 DNA ligase overnight at 16°C, purified and analysed by conventional PCR.

### Locked nucleic acid (LNA) knockdown of miR-221 and miR-222

LCLs 3A-REV were electroporated with 50 nM of LNA anti-miR-221 oligonucleotide (hsa-miR-221 miRCURY LNA, Exiqon), LNA anti-miR-222 oligonucleotide (hsa-miR-222 miRCURY LNA, Exiqon) or scrambled oligonucleotide (miRCURY LNA microRNA inhibitor control, Exiqon) using a Bio-Rad Gene Pulser I (270V, 960μF). After 48 h, dead cells and debris were removed by layering the cells over 3ml Ficoll-plaque (GE Healthcare). Live cells were then collected washed in PBS and extraction was performed as previously described for Western blotting.

### Ethics statement

The primary human B cells used in this study were isolated from buffy-coat residues purchased from the UK Blood Transfusion Service; these were derived from the blood of anonymous volunteer blood donors. No ethical approval is required.

## Supporting Information

S1 Table List of antibodies. (DOCX)

S2 Table Taqman MicroRNA assays. (DOCX)

S3 Table Gene expression qRT-PCR primer sequences. (DOCX)

S4 Table ChIP qPCR primer sequences. (DOCX)

S5 Table Chromosome conformation capture primer sequences. (DOCX)

S1 Fig Western blot analysis of the EBNA3 proteins in LCLs used in this study. (**A**) Western blot analysis of EBNA3A expression from four independent LCLs EBNA3A-KO and EBNA3A-REV (D1, D2, D3 and D4). (**B**) Expression of EBNA3C-HT was evaluated by western blot analysis from two p16-null LCL 3CHT (A2 and C1) cultured for 29 days with (+) or without 4HT (-). (**C**) Western blot analysis of EBNA3C-HT expression from p16-null LCL 3CHT established without the presence of 4HT (never HT) or 30 days after 4HT (+HT) was added to culture medium. (**D**) EBNA3B expression in LCL D1, D2, D3 and D4 EBNA3B-KO (3B-KO) and EBNA3B-REV (3B-REV) was evaluated by Western blot. (**E**) EBNA3A-ERT2 expression from EBNA3A-ERT2 LCLs established without the presence of 4HT (never HT) and 28 days after addition of 4HT (+HT) to culture medium evaluated by Western blotting. All blots were probed for γ-tubulin as a control for loading.
(TIF)

S2 Fig Expression of miR-143/miR-145, miR-221/miR-222 and control RNA RNU48 are not affected by the treatment of LCL WT by 4HT. MiR expression, as well as control RNU48, was determined by qPCR using RNA extracted from two independent wild type (B95-8-BAC) LCLs, established from two different donors (D11 and 13), after being treated or not with HT for 30 days.
(TIF)

S3 Fig Control RNAs RNU48 and ALAS1 were unaffected by EBNA3A or EBNA3C whereas *CXCL9/10* and *ADAM28/ADAMDEC1* are repressed by EBNA3A and EBNA3C respectively. (**A**) RNU48 and ALAS1 expression from LCL D1, D2, D3 and D4 EBNA3A-KO and EBNA3A-REV as well as from p16-null LCL 3CHT A2 and C1 cultured for 29 days with (+HT) or without 4HT (Washed). Expression was determined by quantitative PCR (qPCR). (**B**) Expression of the EBNA3A-repressed genes *CXCL9* and *CXCL10* were assessed by qPCR on the EBNA3A-KO and EBNA3A-REV (D1, D2, D3 and D4) cell lines as a control for the presence/absence of functional EBNA3A (**C**) Expression of EBNA3C-repressed genes *ADAM28* and *ADAMDEC1* were also determined by qPCR on the EBNA3C-conditional (p16-null cell lines) cultured with (+HT) or without 4HT (Washed) as a control for the functional EBNA3C.
(TIF)

S4 Fig Comparison of miR-221 and miR-222 level across several independent LCLs. Levels of miR-221 and miR-222 were compared between LCL D1, D2, D3, D4 EBNA3A-KO and-REV, p16-null 3CHT A2 and C1 culture with (+HT) or without HT (Washed), LCL D1, D2, D3, D4 EBNA3B-KO and-REV. MiR expression was normalized to RNU6B and is shown relative to LCL D1 EBNA3A-REV.
(TIF)

S5 Fig EBNA3A represses *CXCL9 and CXCL10* in LCL EBNA3A-ERT2. Expression levels of the well characterised EBNA3A repressed genes *CXCL9* and *CXCL10* were also determined as a control for the inactivation of EBN3A when 4HT was removed from the culture. Expression of control RNAs ALAS1 and RNU48 expression were also determined.
(TIF)

S6 Fig EBNA3A and EBNA3C both up-regulate miR-221 and miR-222 expression after their activation in the conditional LCLs. MiR-221 and miR-222 expression were determined by real time quantitative RT-PCR (qPCR) from EBNA3A-ERT2 LCLs established without the presence of 4HT (never HT) and 28 days after addition of 4HT to culture medium (+HT) and from p16-null LCL 3CHT also established without the presence of 4HT (never HT) or 30 days after 4HT (+HT) was added to culture medium.
(TIF)

S7 Fig EBNA3A and EBNA3C both repress the pri-miR transcript for miR-143/miR-145. The expression level of the non-coding RNA precursor of miR-143/miR-145 was determined (**A**) in EBNA3A-KO and-REV LCLs; (**B**) in EBNA3A-ERT2 LCLs cultured with (+HT) or without 4HT (Washed); (**C**) in p16-null LCLs 3CHT with (+HT) or without 4HT (Washed); (**D**) in LCL EBNA3A-ERT2 (never HT) cultured for 28 days with (+HT) or without 4HT; (**E**) and p16-null LCL 3CHT (never HT) and cultured for 30 days with (+HT) or without 4HT.
(TIF)

S8 Fig Validation of LCL WT, LCL 3A-TAP, LCL 3C-TAP used for ChIP. (**A**) EBNA3A-TAP, EBNA3B and EBNA3C-TAP expression in the cell lines LCL WT, LCL 3A-TAP, LCL 3C-TAP used in ChIP experiment was evaluated by Western blot. The blot was probed for γ-tubulin as a control for loading. (**B**). ChIP analysis using an anti-Flag antibody was performed as in Fig 7. Primers for the Myoglobin promoter (Myo) were used for qPCR as negative control, whereas primers for known EBNA3A/3C binding sites at the *ADAM28/ADAMDEC1* intergenic enhancer (ADAM) and *CtBP2* locus (CTBP2) were used as positive controls of EBNA3 binding. Values represent ratio of chromatin precipitated, after correction for IgG, relative to 2.5% of input.
(TIF)

S9 Fig EBNA3A and EBNA3C do not bind within at least 1Mbp either side of the putative TSS of the pri-miR-143/145. ChIP-seq data at the miR-143/miR-145 cluster genomic locus generated from LCL 3A-TAP and LCL 3C-TAP (Paschos et al., manuscript in preparation) were displayed using UCSC Genome Browser. The non-coding pri-miR-143/145 (called MIR143-HG in the genome browser) as well as miR-143/miR-145 are highlighted by inclusion in a red box.
(TIF)

S10 Fig Expression of the CDKI p27^KIP1^ in LCLs. (**A**) Western blot showing the expression level of p27^KIP1^ from four EBNA3A-KO (KO) LCLs and their revertant equivalents (REV). Below the Western blot is the p27^KIP1^ mRNA expression level. (**B**) As in (A), but from five LCLs EBNA3A-ERT2 conditional cell lines cultured with (+) or without (-) 4HT for ~30 days. (**C**) Western blot showing the expression of p27^KIP1^ protein and qPCR showing the mRNA level corresponding to p27^KIP1^ from two (p16-null) LCL 3CHT cultured with (+) or without (-) 4HT. All the blots were probed for γ-tubulin as a control for loading.
(TIF)

S11 Fig The effect of removing of 4HT from different 3A-ERT2 LCLs on pRb phosphorylation and population growth. (**A**) Western blot showing the expression of hyperphosphorylated Rb (ppRb). γ-tubulin was used as a control for loading. (**B**) A comparison of the population growth rate between four 3A-ERT2 LCLs (line 1–2 being established from donor D11 and line 3–4 donor D13) cultured with (+HT) or without (Washed) 4HT for ~2 months was analysed by counting the number of viable cells every 2–3 days. Total cell numbers were plotted at each time point. As control, two wild-type LCLs from the same background as 3A-ERT2 LCLs (D11 and D13) were treated or not with HT. Data are representative of at least two independent experiments.
(TIF)

S12 Fig p57^KIP2^ co-immunoprecipitates with CDK6. Immunoprecipitation was performed with mouse anti-CDK2 or anti-CDK6 antibodies on extracts from LCL D4 EBNA3A-REV and EBNA3A-KO. A large excess of mouse IgG was used as a control for non-specific binding and precipitates were compared to 10% input after Western blots were probed for p57^KIP2^, CDK2 or CDK6. p57^KIP2^ (arrowed) appears to precipitate with CDK6 but not CDK2. Immunoglobulin chains are indicated and the asterisk indicates an unidentified non-specific protein band.
(TIF)

S13 Fig EBNA3A and EBNA3C binding sites on miR-221/miR-222 locus can also bind multiple transcription factors. ENCODE GM12878 ChIP-seq data at the EBNA3A and EBNA3C binding sites BS1, BS2 (BS2a and BS2b) and BS3 showed multiple transcription factors also bind to those regions (displayed using UCSC Genome Browser). The three EBNA3s binding sites are highlighted by inclusion in a red box.
(TIF)

## References

[1] Young LS, Rickinson AB (2004) Epstein-Barr virus: 40 years on. Nat Rev Cancer 4: 757–768. 15510157 10.1038/nrc1452

[2] Thorley-Lawson DA, Gross A (2004) Persistence of the Epstein-Barr virus and the origins of associated lymphomas. N Engl J Med 350: 1328–1337. 15044644 10.1056/NEJMra032015

[3] Thorley-Lawson DA, Hawkins JB, Tracy SI, Shapiro M (2013) The pathogenesis of Epstein-Barr virus persistent infection. Current opinion in virology. 10.1016/j.coviro.2013.04.005 PMC3789532 23683686

[4] Forte E, Luftig MA (2011) The role of microRNAs in Epstein-Barr virus latency and lytic reactivation. Microbes and infection / Institut Pasteur 13: 1156–1167. 10.1016/j.micinf.2011.07.007 21835261 PMC4911174

[5] Baer R, Bankier AT, Biggin MD, Deininger PL, Farrell PJ, et al (1984) DNA sequence and expression of the B95-8 Epstein-Barr virus genome. Nature 310: 207–211. 6087149 10.1038/310207a0

[6] Hennessy K, Wang F, Bushman EW, Kieff E (1986) Definitive identification of a member of the Epstein-Barr virus nuclear protein 3 family. Proceedings of the National Academy of Sciences of the United States of America 83: 5693–5697. 3016714 10.1073/pnas.83.15.5693 PMC386355

[7] Tomkinson B, Kieff E (1992) Use of second-site homologous recombination to demonstrate that Epstein-Barr virus nuclear protein 3B is not important for lymphocyte infection or growth transformation in vitro. J Virol 66: 2893–2903. 1313908 10.1128/jvi.66.5.2893-2903.1992 PMC241048

[8] Tomkinson B, Kieff E (1992) Second-site homologous recombination in Epstein-Barr virus: insertion of type 1 EBNA 3 genes in place of type 2 has no effect on in vitro infection. Journal of virology 66: 780–789. 1309912 10.1128/jvi.66.2.780-789.1992 PMC240778

[9] Maruo S, Wu Y, Ishikawa S, Kanda T, Iwakiri D, et al (2006) Epstein-Barr virus nuclear protein EBNA3C is required for cell cycle progression and growth maintenance of lymphoblastoid cells. Proc Natl Acad Sci U S A 103: 19500–19505. 17159137 10.1073/pnas.0604919104 PMC1748255

[10] Maruo S, Johannsen E, Illanes D, Cooper A, Kieff E (2003) Epstein-Barr Virus nuclear protein EBNA3A is critical for maintaining lymphoblastoid cell line growth. J Virol 77: 10437–10447. 12970429 10.1128/JVI.77.19.10437-10447.2003 PMC228516

[11] Hertle ML, Popp C, Petermann S, Maier S, Kremmer E, et al (2009) Differential gene expression patterns of EBV infected EBNA-3A positive and negative human B lymphocytes. PLoS Pathog 5: e1000506 10.1371/journal.ppat.1000506 19578441 PMC2700271

[12] Skalska L, White RE, Franz M, Ruhmann M, Allday MJ (2010) Epigenetic repression of p16(INK4A) by latent Epstein-Barr virus requires the interaction of EBNA3A and EBNA3C with CtBP. PLoS pathogens 6: e1000951 10.1371/journal.ppat.1000951 20548956 PMC2883600

[13] White RE, Ramer PC, Naresh KN, Meixlsperger S, Pinaud L, et al (2012) EBNA3B-deficient EBV promotes B cell lymphomagenesis in humanized mice and is found in human tumors. The Journal of clinical investigation 122: 1487–1502. 10.1172/JCI58092 22406538 PMC3314448

[14] White RE, Groves IJ, Turro E, Yee J, Kremmer E, et al (2010) Extensive co-operation between the Epstein-Barr virus EBNA3 proteins in the manipulation of host gene expression and epigenetic chromatin modification. PloS one 5: e13979 10.1371/journal.pone.0013979 21085583 PMC2981562

[15] Robertson ES, Grossman S, Johannsen E, Miller C, Lin J, et al (1995) Epstein-Barr virus nuclear protein 3C modulates transcription through interaction with the sequence-specific DNA-binding protein J kappa. Journal of virology 69: 3108–3116. 7707539 10.1128/jvi.69.5.3108-3116.1995 PMC189012

[16] Robertson ES, Lin J, Kieff E (1996) The amino-terminal domains of Epstein-Barr virus nuclear proteins 3A, 3B, and 3C interact with RBPJ(kappa). Journal of virology 70: 3068–3074. 8627785 10.1128/jvi.70.5.3068-3074.1996 PMC190168

[17] Jimenez-Ramirez C, Brooks AJ, Forshell LP, Yakimchuk K, Zhao B, et al (2006) Epstein-Barr virus EBNA-3C is targeted to and regulates expression from the bidirectional LMP-1/2B promoter. Journal of virology 80: 11200–11208. 16956945 10.1128/JVI.00897-06 PMC1642179

[18] Zhao B, Sample CE (2000) Epstein-barr virus nuclear antigen 3C activates the latent membrane protein 1 promoter in the presence of Epstein-Barr virus nuclear antigen 2 through sequences encompassing an spi-1/Spi-B binding site. Journal of virology 74: 5151–5160. 10799590 10.1128/jvi.74.11.5151-5160.2000 PMC110868

[19] Jiang S, Willox B, Zhou H, Holthaus AM, Wang A, et al (2014) Epstein-Barr virus nuclear antigen 3C binds to BATF/IRF4 or SPI1/IRF4 composite sites and recruits Sin3A to repress CDKN2A. Proceedings of the National Academy of Sciences of the United States of America 111: 421–426. 10.1073/pnas.1321704111 24344258 PMC3890834

[20] McClellan MJ, Wood CD, Ojeniyi O, Cooper TJ, Kanhere A, et al (2013) Modulation of enhancer looping and differential gene targeting by Epstein-Barr virus transcription factors directs cellular reprogramming. PLoS pathogens 9: e1003636 10.1371/journal.ppat.1003636 24068937 PMC3771879

[21] Harth-Hertle ML, Scholz BA, Erhard F, Glaser LV, Dolken L, et al (2013) Inactivation of intergenic enhancers by EBNA3A initiates and maintains polycomb signatures across a chromatin domain encoding CXCL10 and CXCL9. PLoS pathogens 9: e1003638 10.1371/journal.ppat.1003638 24068939 PMC3777872

[22] Cotter MA 2nd, Robertson ES (2000) Modulation of histone acetyltransferase activity through interaction of epstein-barr nuclear antigen 3C with prothymosin alpha. Molecular and cellular biology 20: 5722–5735. 10891508 10.1128/mcb.20.15.5722-5735.2000 PMC86050

[23] Hickabottom M, Parker GA, Freemont P, Crook T, Allday MJ (2002) Two nonconsensus sites in the Epstein-Barr virus oncoprotein EBNA3A cooperate to bind the co-repressor carboxyl-terminal-binding protein (CtBP). J Biol Chem 277: 47197–47204. 12372828 10.1074/jbc.M208116200

[24] Knight JS, Lan K, Subramanian C, Robertson ES (2003) Epstein-Barr virus nuclear antigen 3C recruits histone deacetylase activity and associates with the corepressors mSin3A and NCoR in human B-cell lines. J Virol 77: 4261–4272. 12634383 10.1128/JVI.77.7.4261-4272.2003 PMC150657

[25] Radkov SA, Touitou R, Brehm A, Rowe M, West M, et al (1999) Epstein-Barr virus nuclear antigen 3C interacts with histone deacetylase to repress transcription. J Virol 73: 5688–5697. 10364319 10.1128/jvi.73.7.5688-5697.1999 PMC112628

[26] Touitou R, Hickabottom M, Parker G, Crook T, Allday MJ (2001) Physical and functional interactions between the corepressor CtBP and the Epstein-Barr virus nuclear antigen EBNA3C. J Virol 75: 7749–7755. 11462050 10.1128/JVI.75.16.7749-7755.2001 PMC115013

[27] Paschos K, Parker GA, Watanatanasup E, White RE, Allday MJ (2012) BIM promoter directly targeted by EBNA3C in polycomb-mediated repression by EBV. Nucleic acids research 40: 7233–7246. 10.1093/nar/gks391 22584624 PMC3424555

[28] McClellan MJ, Khasnis S, Wood CD, Palermo RD, Schlick SN, et al (2012) Downregulation of integrin receptor-signaling genes by Epstein-Barr virus EBNA 3C via promoter-proximal and-distal binding elements. Journal of virology 86: 5165–5178. 10.1128/JVI.07161-11 22357270 PMC3347391

[29] Schmidt SC, Jiang S, Zhou H, Willox B, Holthaus AM, et al (2015) Epstein-Barr virus nuclear antigen 3A partially coincides with EBNA3C genome-wide and is tethered to DNA through BATF complexes. Proceedings of the National Academy of Sciences of the United States of America 112: 554–559. 10.1073/pnas.1422580112 25540416 PMC4299249

[30] Skalska L, White RE, Parker GA, Sinclair AJ, Paschos K, et al (2013) Induction of p16(INK4a) is the major barrier to proliferation when Epstein-Barr virus (EBV) transforms primary B cells into lymphoblastoid cell lines. PLoS pathogens 9: e1003187 10.1371/journal.ppat.1003187 23436997 PMC3578823

[31] Allday MJ (2013) EBV finds a polycomb-mediated, epigenetic solution to the problem of oncogenic stress responses triggered by infection. Frontiers in genetics 4: 212 10.3389/fgene.2013.00212 24167519 PMC3807040

[32] Bazot Q, Deschamps T, Tafforeau L, Siouda M, Leblanc P, et al (2014) Epstein-Barr virus nuclear antigen 3A protein regulates CDKN2B transcription via interaction with MIZ-1. Nucleic acids research 42: 9700–9716. 10.1093/nar/gku697 25092922 PMC4150796

[33] Fabian MR, Sonenberg N, Filipowicz W (2010) Regulation of mRNA translation and stability by microRNAs. Annual review of biochemistry 79: 351–379. 10.1146/annurev-biochem-060308-103103 20533884

[34] Wilczynska A, Bushell M (2015) The complexity of miRNA-mediated repression. Cell death and differentiation 22: 22–33. 10.1038/cdd.2014.112 25190144 PMC4262769

[35] Lim LP, Lau NC, Garrett-Engele P, Grimson A, Schelter JM, et al (2005) Microarray analysis shows that some microRNAs downregulate large numbers of target mRNAs. Nature 433: 769–773. 15685193 10.1038/nature03315

[36] Calin GA, Croce CM (2006) MicroRNA signatures in human cancers. Nature reviews Cancer 6: 857–866. 17060945 10.1038/nrc1997

[37] Esquela-Kerscher A, Slack FJ (2006) Oncomirs—microRNAs with a role in cancer. Nature reviews Cancer 6: 259–269. 16557279 10.1038/nrc1840

[38] Feldman ER, Kara M, Coleman CB, Grau KR, Oko LM, et al (2014) Virus-encoded microRNAs facilitate gammaherpesvirus latency and pathogenesis in vivo. mBio 5: e00981–00914. 10.1128/mBio.00981-14 24865551 PMC4045068

[39] Zhao Y, Xu H, Yao Y, Smith LP, Kgosana L, et al (2011) Critical role of the virus-encoded microRNA-155 ortholog in the induction of Marek's disease lymphomas. PLoS pathogens 7: e1001305 10.1371/journal.ppat.1001305 21383974 PMC3044692

[40] Kuzembayeva M, Hayes M, Sugden B (2014) Multiple functions are mediated by the miRNAs of Epstein-Barr virus. Current opinion in virology 7: 61–65. 10.1016/j.coviro.2014.04.003 24814666 PMC4149930

[41] Moody R, Zhu Y, Huang Y, Cui X, Jones T, et al (2013) KSHV microRNAs mediate cellular transformation and tumorigenesis by redundantly targeting cell growth and survival pathways. PLoS pathogens 9: e1003857 10.1371/journal.ppat.1003857 24385912 PMC3873467

[42] Linnstaedt SD, Gottwein E, Skalsky RL, Luftig MA, Cullen BR (2010) Virally induced cellular microRNA miR-155 plays a key role in B-cell immortalization by Epstein-Barr virus. Journal of virology 84: 11670–11678. 10.1128/JVI.01248-10 20844043 PMC2977875

[43] Sun T, Yang M, Kantoff P, Lee GS (2009) Role of microRNA-221/-222 in cancer development and progression. Cell cycle 8: 2315–2316. 19625765 10.4161/cc.8.15.9221

[44] Pallante P, Visone R, Ferracin M, Ferraro A, Berlingieri MT, et al (2006) MicroRNA deregulation in human thyroid papillary carcinomas. Endocrine-related cancer 13: 497–508. 16728577 10.1677/erc.1.01209

[45] Ciafre SA, Galardi S, Mangiola A, Ferracin M, Liu CG, et al (2005) Extensive modulation of a set of microRNAs in primary glioblastoma. Biochemical and biophysical research communications 334: 1351–1358. 16039986 10.1016/j.bbrc.2005.07.030

[46] Galardi S, Mercatelli N, Giorda E, Massalini S, Frajese GV, et al (2007) miR-221 and miR-222 expression affects the proliferation potential of human prostate carcinoma cell lines by targeting p27Kip1. The Journal of biological chemistry 282: 23716–23724. 17569667 10.1074/jbc.M701805200

[47] Galardi S, Mercatelli N, Farace MG, Ciafre SA (2011) NF-kB and c-Jun induce the expression of the oncogenic miR-221 and miR-222 in prostate carcinoma and glioblastoma cells. Nucleic acids research 39: 3892–3902. 10.1093/nar/gkr006 21245048 PMC3089483

[48] Gottardo F, Liu CG, Ferracin M, Calin GA, Fassan M, et al (2007) Micro-RNA profiling in kidney and bladder cancers. Urologic oncology 25: 387–392. 17826655 10.1016/j.urolonc.2007.01.019

[49] Lee EJ, Gusev Y, Jiang J, Nuovo GJ, Lerner MR, et al (2007) Expression profiling identifies microRNA signature in pancreatic cancer. International journal of cancer Journal international du cancer 120: 1046–1054. 17149698 10.1002/ijc.22394 PMC2680248

[50] Pineau P, Volinia S, McJunkin K, Marchio A, Battiston C, et al (2010) miR-221 overexpression contributes to liver tumorigenesis. Proceedings of the National Academy of Sciences of the United States of America 107: 264–269. 10.1073/pnas.0907904107 20018759 PMC2806773

[51] Rommer A, Steinleitner K, Hackl H, Schneckenleithner C, Engelmann M, et al (2013) Overexpression of primary microRNA 221/222 in acute myeloid leukemia. BMC cancer 13: 364 10.1186/1471-2407-13-364 23895238 PMC3733744

[52] Lawrie CH, Soneji S, Marafioti T, Cooper CD, Palazzo S, et al (2007) MicroRNA expression distinguishes between germinal center B cell-like and activated B cell-like subtypes of diffuse large B cell lymphoma. International journal of cancer Journal international du cancer 121: 1156–1161. 17487835 10.1002/ijc.22800

[53] Malumbres R, Sarosiek KA, Cubedo E, Ruiz JW, Jiang X, et al (2009) Differentiation stage-specific expression of microRNAs in B lymphocytes and diffuse large B-cell lymphomas. Blood 113: 3754–3764. 10.1182/blood-2008-10-184077 19047678 PMC2670792

[54] Andrade TA, Evangelista AF, Campos AH, Poles WA, Borges NM, et al (2014) A microRNA signature profile in EBV+ diffuse large B-cell lymphoma of the elderly. Oncotarget 5: 11813–11826. 25544772 10.18632/oncotarget.2952 PMC4322989

[55] Wang J, Liu S, Sun GP, Wang F, Zou YF, et al (2014) Prognostic significance of microRNA-221/222 expression in cancers: evidence from 1,204 subjects. The International journal of biological markers 29: e129–141. 10.5301/jbm.5000058 24474451

[56] Visone R, Russo L, Pallante P, De Martino I, Ferraro A, et al (2007) MicroRNAs (miR)-221 and miR-222, both overexpressed in human thyroid papillary carcinomas, regulate p27Kip1 protein levels and cell cycle. Endocrine-related cancer 14: 791–798. 17914108 10.1677/ERC-07-0129

[57] Fornari F, Gramantieri L, Ferracin M, Veronese A, Sabbioni S, et al (2008) MiR-221 controls CDKN1C/p57 and CDKN1B/p27 expression in human hepatocellular carcinoma. Oncogene 27: 5651–5661. 10.1038/onc.2008.178 18521080

[58] le Sage C, Nagel R, Egan DA, Schrier M, Mesman E, et al (2007) Regulation of the p27(Kip1) tumor suppressor by miR-221 and miR-222 promotes cancer cell proliferation. The EMBO journal 26: 3699–3708. 17627278 10.1038/sj.emboj.7601790 PMC1949005

[59] Medina R, Zaidi SK, Liu CG, Stein JL, van Wijnen AJ, et al (2008) MicroRNAs 221 and 222 bypass quiescence and compromise cell survival. Cancer research 68: 2773–2780. 10.1158/0008-5472.CAN-07-6754 18413744 PMC3613850

[60] Elia L, Quintavalle M, Zhang J, Contu R, Cossu L, et al (2009) The knockout of miR-143 and -145 alters smooth muscle cell maintenance and vascular homeostasis in mice: correlates with human disease. Cell death and differentiation 16: 1590–1598. 10.1038/cdd.2009.153 19816508 PMC3014107

[61] Kent OA, Chivukula RR, Mullendore M, Wentzel EA, Feldmann G, et al (2010) Repression of the miR-143/145 cluster by oncogenic Ras initiates a tumor-promoting feed-forward pathway. Genes & development 24: 2754–2759. 21159816 10.1101/gad.1950610 PMC3003192

[62] Bonifacio LN, Jarstfer MB (2010) MiRNA profile associated with replicative senescence, extended cell culture, and ectopic telomerase expression in human foreskin fibroblasts. PloS one 5. 10.1371/journal.pone.0012519 PMC2931704 20824140

[63] Iio A, Nakagawa Y, Hirata I, Naoe T, Akao Y (2010) Identification of non-coding RNAs embracing microRNA-143/145 cluster. Molecular cancer 9: 136 10.1186/1476-4598-9-136 20525177 PMC2903500

[64] Takagi T, Iio A, Nakagawa Y, Naoe T, Tanigawa N, et al (2009) Decreased expression of microRNA-143 and -145 in human gastric cancers. Oncology 77: 12–21. 10.1159/000218166 19439999

[65] Akao Y, Nakagawa Y, Hirata I, Iio A, Itoh T, et al (2010) Role of anti-oncomirs miR-143 and -145 in human colorectal tumors. Cancer gene therapy 17: 398–408. 10.1038/cgt.2009.88 20094072

[66] Lui WO, Pourmand N, Patterson BK, Fire A (2007) Patterns of known and novel small RNAs in human cervical cancer. Cancer research 67: 6031–6043. 17616659 10.1158/0008-5472.CAN-06-0561

[67] Vosa U, Vooder T, Kolde R, Vilo J, Metspalu A, et al (2013) Meta-analysis of microRNA expression in lung cancer. International journal of cancer Journal international du cancer 132: 2884–2893. 10.1002/ijc.27981 23225545

[68] Zhou F, Li S, Meng HM, Qi LQ, Gu L (2013) MicroRNA and histopathological characterization of pure mucinous breast carcinoma. Cancer biology & medicine 10: 22–27. 23691441 10.7497/j.issn.2095-3941.2013.01.004 PMC3643684

[69] Chen HC, Chen GH, Chen YH, Liao WL, Liu CY, et al (2009) MicroRNA deregulation and pathway alterations in nasopharyngeal carcinoma. British journal of cancer 100: 1002–1011. 10.1038/sj.bjc.6604948 19293812 PMC2661776

[70] Yoshino H, Seki N, Itesako T, Chiyomaru T, Nakagawa M, et al (2013) Aberrant expression of microRNAs in bladder cancer. Nature reviews Urology 10: 396–404. 10.1038/nrurol.2013.113 23712207

[71] Szczyrba J, Loprich E, Wach S, Jung V, Unteregger G, et al (2010) The microRNA profile of prostate carcinoma obtained by deep sequencing. Molecular cancer research: MCR 8: 529–538. 10.1158/1541-7786.MCR-09-0443 20353999

[72] Iorio MV, Visone R, Di Leva G, Donati V, Petrocca F, et al (2007) MicroRNA signatures in human ovarian cancer. Cancer research 67: 8699–8707. 17875710 10.1158/0008-5472.CAN-07-1936

[73] Xing AY, Wang B, Shi DB, Zhang XF, Gao C, et al (2013) Deregulated expression of miR-145 in manifold human cancer cells. Experimental and molecular pathology 95: 91–97. 10.1016/j.yexmp.2013.05.003 23714355

[74] Akao Y, Nakagawa Y, Kitade Y, Kinoshita T, Naoe T (2007) Downregulation of microRNAs-143 and -145 in B-cell malignancies. Cancer science 98: 1914–1920. 17892514 10.1111/j.1349-7006.2007.00618.x PMC11158757

[75] Rangrez AY, Massy ZA, Metzinger-Le Meuth V, Metzinger L (2011) miR-143 and miR-145: molecular keys to switch the phenotype of vascular smooth muscle cells. Circulation Cardiovascular genetics 4: 197–205. 10.1161/CIRCGENETICS.110.958702 21505201

[76] Zhang C, Zhang J, Zhang A, Wang Y, Han L, et al (2010) PUMA is a novel target of miR-221/222 in human epithelial cancers. International journal of oncology 37: 1621–1626. 21042732 10.3892/ijo_00000816

[77] Phatnani HP, Greenleaf AL (2006) Phosphorylation and functions of the RNA polymerase II CTD. Genes & development 20: 2922–2936. 17079683 10.1101/gad.1477006

[78] Maruo S, Zhao B, Johannsen E, Kieff E, Zou J, et al (2011) Epstein-Barr virus nuclear antigens 3C and 3A maintain lymphoblastoid cell growth by repressing p16INK4A and p14ARF expression. Proceedings of the National Academy of Sciences of the United States of America 108: 1919–1924. 10.1073/pnas.1019599108 21245331 PMC3033265

[79] Pombo A, Dillon N (2015) Three-dimensional genome architecture: players and mechanisms. Nature reviews Molecular cell biology 16: 245–257. 10.1038/nrm3965 25757416

[80] Hutzinger R, Mrazek J, Vorwerk S, Huttenhofer A (2010) NcRNA-microchip analysis: a novel approach to identify differential expression of noncoding RNAs. RNA biology 7: 586–595. 21037422 10.4161/rna.7.5.12971 PMC3073255

[81] Cameron JE, Fewell C, Yin Q, McBride J, Wang X, et al (2008) Epstein-Barr virus growth/latency III program alters cellular microRNA expression. Virology 382: 257–266. 10.1016/j.virol.2008.09.018 18950829 PMC2640950

[82] Cameron JE, Yin Q, Fewell C, Lacey M, McBride J, et al (2008) Epstein-Barr virus latent membrane protein 1 induces cellular MicroRNA miR-146a, a modulator of lymphocyte signaling pathways. Journal of virology 82: 1946–1958. 18057241 10.1128/JVI.02136-07 PMC2258704

[83] Gunasekharan V, Laimins LA (2013) Human papillomaviruses modulate microRNA 145 expression to directly control genome amplification. Journal of virology 87: 6037–6043. 10.1128/JVI.00153-13 23468503 PMC3648148

[84] Godshalk SE, Bhaduri-McIntosh S, Slack FJ (2008) Epstein-Barr virus-mediated dysregulation of human microRNA expression. Cell cycle 7: 3595–3600. 19001862 10.4161/cc.7.22.7120

[85] Tursiella ML, Bowman ER, Wanzeck KC, Throm RE, Liao J, et al (2014) Epstein-Barr Virus Nuclear Antigen 3A Promotes Cellular Proliferation by Repression of the Cyclin-Dependent Kinase Inhibitor p21WAF1/CIP1. PLoS pathogens 10: e1004415 10.1371/journal.ppat.1004415 25275486 PMC4183747

[86] Banerjee S, Lu J, Cai Q, Sun Z, Jha HC, et al (2014) EBNA3C augments Pim-1 mediated phosphorylation and degradation of p21 to promote B-cell proliferation. PLoS pathogens 10: e1004304 10.1371/journal.ppat.1004304 25121590 PMC4133388

[87] Sherr CJ (2012) Ink4-Arf Locus in Cancer and Aging. Wiley interdisciplinary reviews Developmental biology 1: 731–741. 22960768 10.1002/wdev.40 PMC3434949

[88] Kelly G, Bell A, Rickinson A (2002) Epstein-Barr virus-associated Burkitt lymphomagenesis selects for downregulation of the nuclear antigen EBNA2. Nat Med 8: 1098–1104. 12219084 10.1038/nm758

[89] Feil R, Wagner J, Metzger D, Chambon P (1997) Regulation of Cre recombinase activity by mutated estrogen receptor ligand-binding domains. Biochemical and biophysical research communications 237: 752–757. 9299439 10.1006/bbrc.1997.7124

[90] Delecluse HJ, Hilsendegen T, Pich D, Zeidler R, Hammerschmidt W (1998) Propagation and recovery of intact, infectious Epstein-Barr virus from prokaryotic to human cells. Proc Natl Acad Sci U S A 95: 8245–8250. 9653172 10.1073/pnas.95.14.8245 PMC20961

[91] Anderton E, Yee J, Smith P, Crook T, White RE, et al (2008) Two Epstein-Barr virus (EBV) oncoproteins cooperate to repress expression of the proapoptotic tumour-suppressor Bim: clues to the pathogenesis of Burkitt's lymphoma. Oncogene 27: 421–433. 17653091 10.1038/sj.onc.1210668

[92] Hagege H, Klous P, Braem C, Splinter E, Dekker J, et al (2007) Quantitative analysis of chromosome conformation capture assays (3C-qPCR). Nature protocols 2: 1722–1733. 17641637 10.1038/nprot.2007.243

[93] Tempera I, Klichinsky M, Lieberman PM (2011) EBV latency types adopt alternative chromatin conformations. PLoS pathogens 7: e1002180 10.1371/journal.ppat.1002180 21829357 PMC3145795

