# Supplementary material for: Epstein-Barr Virus Proteins EBNA3A and EBNA3C Together Induce Expression of the Oncogenic MicroRNA Cluster miR-221/miR-222 and Ablate Expression of Its Target p57KIP2
Source: PLoS Pathog. 2015 Jul 8;11(7):e1005031. doi: 10.1371/journal.ppat.1005031 (PMC4496050; doi:10.1371/journal.ppat.1005031)
Supplement: S1 Table — (DOCX) [file ppat.1005031.s001.docx]

| Antibody | Application | Description | Source |
| --- | --- | --- | --- |
| γ-tubulin | WB | Mouse monoclonal IgG_1_ | Sigma, T6557 |
| EBNA1 | WB | Human serum | Gift from Prof Paul Farrell |
| EBNA2 | WB | Mouse monoclonal IgG_1_ | Abcam, ab90543 |
| EBNA3A | WB | Sheep polyclonal | Abcam, ab16126 |
| EBNA3B | WB | 6C9 Rat monoclonal | (White et al., 2010) |
| EBNA3C | WB | A-10 hybridoma  Mouse monoclonal | Gift from Prof Martin Rowe  (Maunders et al., 1994) |
| EBNA-LP | WB | JF-186  Mouse monoclonal | (Finke et al., 1987) |
| LMP1 | WB | Mouse monoclonal IgG_1_ | DAKO, CS1-4 |
| p21^CIP1^ | WB | Rabbit monoclonal | Cell Signaling, 2947S |
| p27^KIP1^ | WB | Mouse monoclonal IgG_1_ | BD Transductions, 610242 |
| p57^KIP2^ | WB | Rabbit monoclonal | Abcam, ab133531 |
| PUMA | WB | Rabbit polyclonal | Sigma, P4618 |
| Phospho-Rb | WB | Rabbit polyclonal | Cell Signaling, 9308S |
| Rb | WB | Mouse monoclonal IgG_1_ | BD Transductions, 554136 |
| CDK2 | IP | Mouse monoclonal IgG_1_ | Santa Cruz, sc-6248 |
| CDK2 | WB | Rabbit polyclonal | Santa Cruz, sc-163 |
| CDK6 | IP | Mouse monoclonal IgG_1_ | Santa Cruz, sc-7961 |
| CDK6 | WB | Rabbit polyclonal | Santa Cruz, sc-177 |
| DYKDDDDK Tag | ChIP | Rabbit polyclonal | Cell Signaling, 2368S |
| H3K4me3 | ChIP | Rabbit polyclonal | Millipore, 17-614 |
| H3K9ac | ChIP | Rabbit polyclonal | Millipore 17-658 |
| H3K27ac | ChIP | Mouse monoclonal IgG_1_ | Millipore, 17-683 |
| Total RNA pol II | ChIP | Rabbit polyclonal | Santa Cruz, sc-899 |
| RNA pol II CTD repeat YSPTSPS (phospho S5) | ChIP | Rabbit polyclonal | Abcam, ab5131 |

**S1 Table. List of Antibodies used in the study**
