# Supplementary material for: Epstein-Barr Virus Proteins EBNA3A and EBNA3C Together Induce Expression of the Oncogenic MicroRNA Cluster miR-221/miR-222 and Ablate Expression of Its Target p57KIP2
Source: PLoS Pathog. 2015 Jul 8;11(7):e1005031. doi: 10.1371/journal.ppat.1005031 (PMC4496050; doi:10.1371/journal.ppat.1005031)
Supplement: S2 Table — (DOCX) [file ppat.1005031.s002.docx]

| Assay | ID |
| --- | --- |
| miR-143 | 002249 |
| miR-145 | 002278 |
| miR-221 | 000524 |
| miR-222 | 002276 |
| RNU6B | 001093 |
| RNU48 | 001006 |

**S2 Table. TaqMan MicroRNA Assay used in the study.**
