# Supplementary material for: Epstein-Barr Virus Proteins EBNA3A and EBNA3C Together Induce Expression of the Oncogenic MicroRNA Cluster miR-221/miR-222 and Ablate Expression of Its Target p57KIP2
Source: PLoS Pathog. 2015 Jul 8;11(7):e1005031. doi: 10.1371/journal.ppat.1005031 (PMC4496050; doi:10.1371/journal.ppat.1005031)
Supplement: S3 Table — (DOCX) [file ppat.1005031.s003.docx]

| Target Gene | Sequence (5'🡪3') | Reference |
| --- | --- | --- |
| ALAS1 | F-TCCACTGCAGCAGTACACTACCA | (Skalska et al., 2010) |
|  | R-ACGGAAGCTGTGTGCCATCT |  |
| ADAM 28 | F-GTTGCAGGGACAATGGCACA | ND |
|  | R-TGAGACGGCTGCAGGAACTG |  |
| ADAMDEC1 | F-CCTTGGTATGCCTGATGTTCCA | ND |
|  | R-CAGCAGGCACTTTGGTTTCTGA |  |
| CXCL9 | F- GTAGTGAGAAAGGGTCGCTGT | ND |
|  | R- AGGGCTTGGGGCAAATTGTT |  |
| CXCL10 | F- CCTGCAAGCCAATTTTGTCCACGTGT | ND |
|  | R- AGCACTGCATCGATTTTGCTCCCCTC |  |
| GNB2L1 | F-GCTTGCAGTTAGCCAGGTTC | (Skalska et al., 2010) |
|  | R-GAGTGTGGCCTTCTCCTCTG |  |
| p27 | F-GGAGCAATGCGCAGGAATAA | (Lindberg et al., 2008) |
|  | R-TGGGGAACCGTCTGAAACAT |  |
| p57 | F- GGCGATCAAGAAGCTGTCC | ND |
|  | R-GACTTCTCAGGCGCTGATCT |  |
| pri-miR-143/145 | F-AGCAAGAACTCTGGAGAAGCA | (Iio et al., 2013) |
|  | R-GAGAGGCGTGGGTGAGAG |  |
| pri-miR-221/222 | F- ACTTGCCCTCCTTTCCTTTC | (Rommer et al., 2013) |
|  | R- AGGTGTTTCCGACGCATTAC |  |

**S3 Table. List of primers used to assay cDNA from mRNA transcripts**

ND: Newly designed.
