# Supplementary material for: Epstein-Barr Virus Proteins EBNA3A and EBNA3C Together Induce Expression of the Oncogenic MicroRNA Cluster miR-221/miR-222 and Ablate Expression of Its Target p57KIP2
Source: PLoS Pathog. 2015 Jul 8;11(7):e1005031. doi: 10.1371/journal.ppat.1005031 (PMC4496050; doi:10.1371/journal.ppat.1005031)
Supplement: S4 Table — (DOCX) [file ppat.1005031.s004.docx]

| Target | Primer Name | Sequence (5'🡪3') | Reference |
| --- | --- | --- | --- |
| miR-221/-222 | BS1 | F-AGAGCTTTACTCCAGAAGGCCACAG | ND |
|  |  | R-GGGAGTCTGGGAAGGCCCTAAT |  |
|  | TSS (2kb) | F-TCCAGCACCTAAGAAAATATGTGGC | ND |
|  |  | R-CCCATGTACGTAATTTTAAACAACCTC |  |
|  | EnhA | F-ATCTTGTGCCAACCAGTCCCTTCT | (Galardi et al., 2011) |
|  |  | R-AACATTCTCTGGGACGTTCCTGCT |  |
|  | BS2a | F-TGGTCCTTCCTCTAAGATTGGTCC | (Galardi et al., 2011) |
|  |  | R-ACCAAACTGCAGGAACTGACTCAT |  |
|  | BS2b | F-GAGCAAACTAAGCTCTCCTCCACACA | ND |
|  |  | R-CGCCTTTGGCTGAAACTGACTG |  |
|  | BS3 | F-ATGAGATCAACTGAAAACAAGGAATTCC | ND |
|  |  | R-CACAAATCAACGGGAACAGTCT |  |
|  | C1 | F-CCCTTCAAATTTCATACATGCT | ND |
|  |  | R-ACATAAAATCAGAATGGGCT |  |
|  | TSS (28kb) | F-GTTGAGCAGCAGCCCAGGCA | ND |
|  |  | R-TGCTGGCTGCGATTGGTCAG |  |
|  | C2 | F-AATATGGGCTCAACCTCATCCTTCAA | ND |
|  |  | R-AAATTGGGAATGGCAAGCGG |  |
|  | C3 | F-GGACATTGGAAGTGTCATCAGCCA | ND |
|  |  | R-TCAAGGGCAGTGCTCTGTCC |  |
| Myoglobin | Myo | F-GGAGAAAGAAGGGGAATCACA | (Paschos et al., 2012) |
|  |  | R-GATAAATATAGCCAACGCCACA |  |
| ADAM  gene cluster | ADAM | F-CCTATGTCTCGCTTCCTGCT | (McClellan et al., 2012) |
|  |  | F-CTTCATGGCTACAGACTCTTGG |  |
| ADAM28 | ADAM28 TSS | F-CCATTGTTGCAGGACCACAG | ND |
|  |  | R-GCCTCCTCTCCAGTGAGACA |  |
| CTBP2 | CTBP2 | F- TTGCATATTTGGGATTTCAGTTT | (McClellan et al., 2013) |
|  |  | R-TTCTGTGTGAAACAGTTGTGGTT |  |
| CXCL10 | CXCL10 TSS | F- TCCCTCCCTAATTCTGATTGG | (Harth-Hertle et al., 2013) |
|  |  | R- AGCAGAGGGAAATTCCGTAAC |  |

**S4 Table. List of primers used to analyze precipitated DNA from ChIP**

ND: Newly designed
