# Supplementary material for: Epstein-Barr Virus Proteins EBNA3A and EBNA3C Together Induce Expression of the Oncogenic MicroRNA Cluster miR-221/miR-222 and Ablate Expression of Its Target p57KIP2
Source: PLoS Pathog. 2015 Jul 8;11(7):e1005031. doi: 10.1371/journal.ppat.1005031 (PMC4496050; doi:10.1371/journal.ppat.1005031)
Supplement: S5 Table — (DOCX) [file ppat.1005031.s005.docx]

| Primer Name | Sequence (5'🡪3') |
| --- | --- |
| P to BS2 | F-TCTGAGAACTATATGAAGCTGTTTCAAG |
|  | R-GACTTTATTCACTGCTGTATTACCAGAC |
| P to BS3 | F-TCTGAGAACTATATGAAGCTGTTTCAAG |
|  | R-AGCTTAAAGCACAAATCTACTTATTTCC |
| P to NC | F-TCTGAGAACTATATGAAGCTGTTTCAAG |
|  | R-AAAGGTCTCTCTATGCCATAATACATTT |
| L1 | F-AAAAATACCCAAGATCCACTGTTTATT |
| L2 | R-TTGAAACAGCTTCATATAGTTCTCAGAT |

**S5 Table. List of primers used for Chromosome Conformation Capture assay**
