# Supplementary figures and images for: Epstein-Barr Virus Proteins EBNA3A and EBNA3C Together Induce Expression of the Oncogenic MicroRNA Cluster miR-221/miR-222 and Ablate Expression of Its Target p57KIP2
Source: PLoS Pathog. 2015 Jul 8;11(7):e1005031. doi: 10.1371/journal.ppat.1005031 (PMC4496050; doi:10.1371/journal.ppat.1005031)

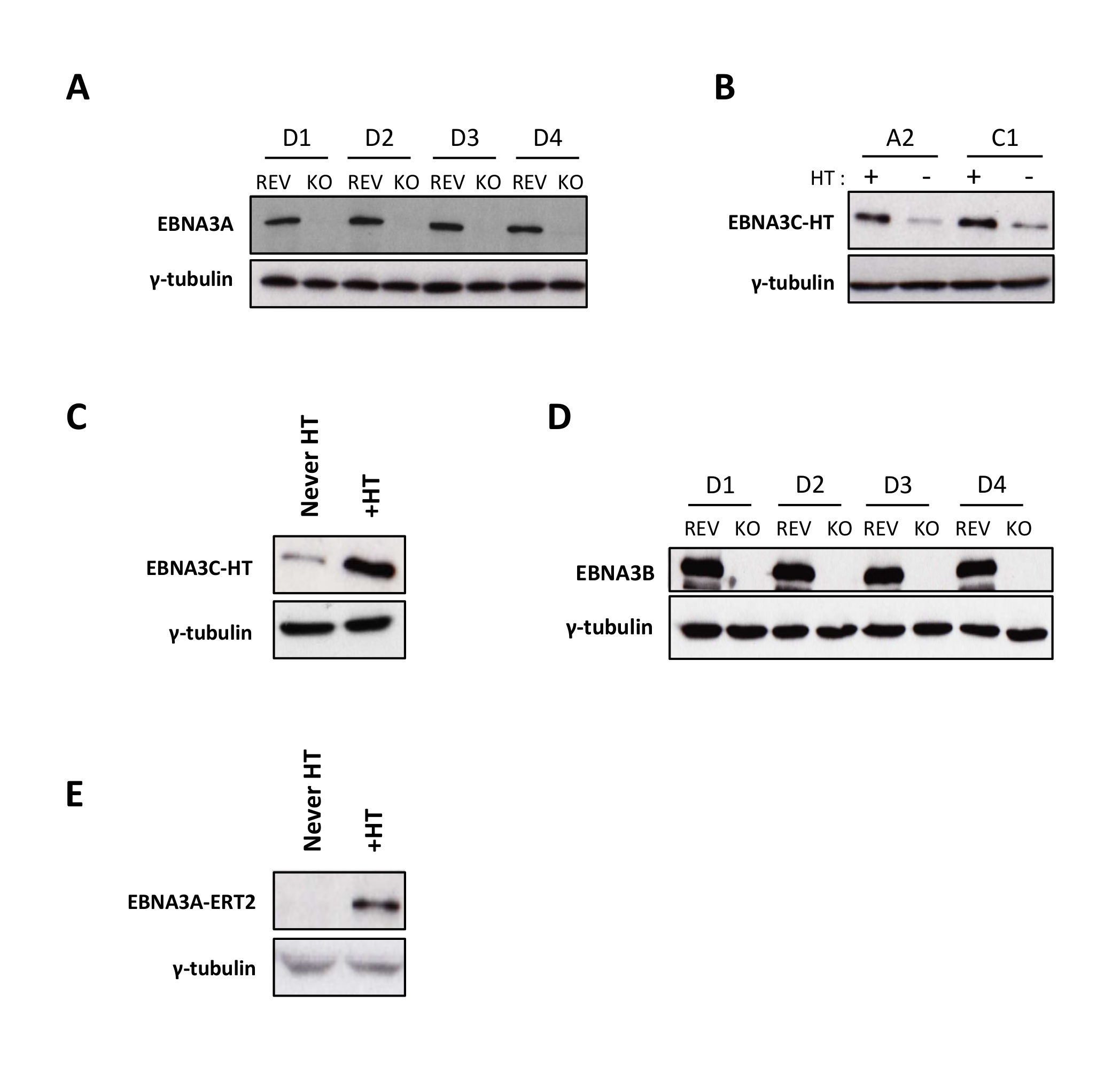

Supplement: S1 Fig — (A) Western blot analysis of EBNA3A expression from four independent LCLs EBNA3A-KO and EBNA3A-REV (D1, D2, D3 and D4). (B) Expression of EBNA3C-HT was evaluated by western blot analysis from two p16-null LCL 3CHT (A2 and C1) cultured for 29 days with (+) or without 4HT (-). (C) Western blot analysis of EBNA3C-HT expression from p16-null LCL 3CHT established without the presence of 4HT (never HT) or 30 days after 4HT (+HT) was added to culture medium. (D) EBNA3B expression in LCL D1, D2, D3 and D4 EBNA3B-KO (3B-KO) and EBNA3B-REV (3B-REV) was evaluated by Western blot. (E) EBNA3A-ERT2 expression from EBNA3A-ERT2 LCLs established without the presence of 4HT (never HT) and 28 days after addition of 4HT (+HT) to culture medium evaluated by Western blotting. All blots were probed for γ-tubulin as a control for loading. (TIF) [file ppat.1005031.s006.tif]

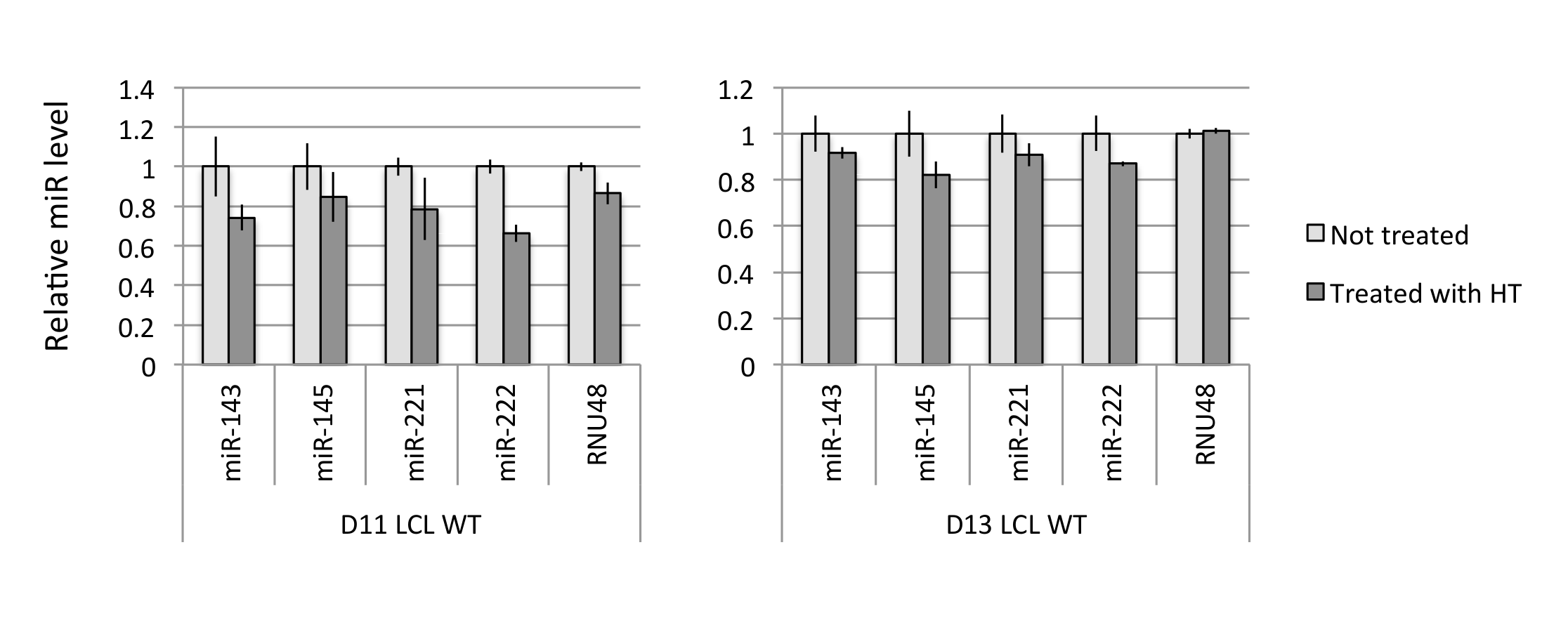

Supplement: S2 Fig — MiR expression, as well as control RNU48, was determined by qPCR using RNA extracted from two independent wild type (B95-8-BAC) LCLs, established from two different donors (D11 and 13), after being treated or not with HT for 30 days. (TIF) [file ppat.1005031.s007.tif]

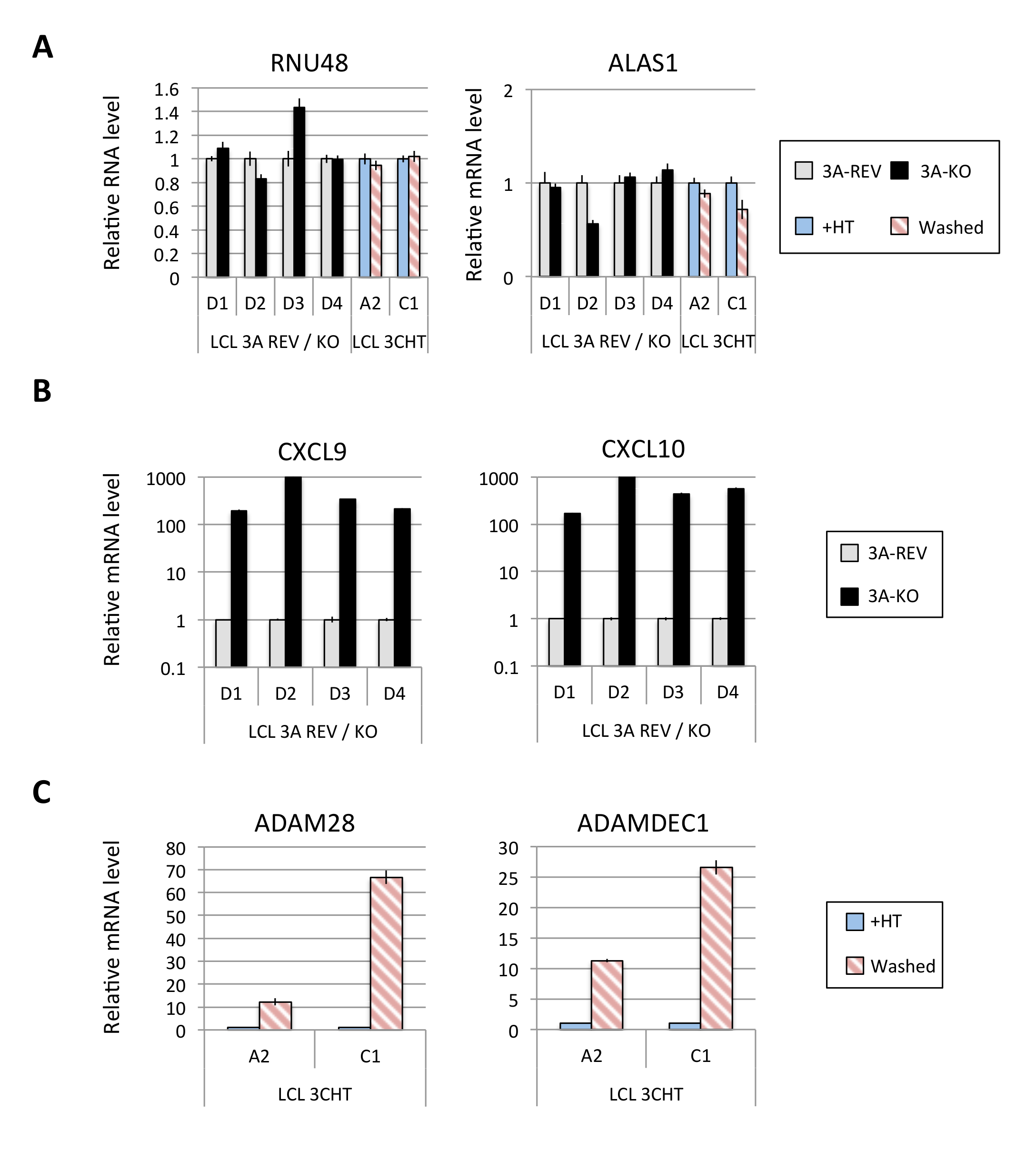

Supplement: S3 Fig — (A) RNU48 and ALAS1 expression from LCL D1, D2, D3 and D4 EBNA3A-KO and EBNA3A-REV as well as from p16-null LCL 3CHT A2 and C1 cultured for 29 days with (+HT) or without 4HT (Washed). Expression was determined by quantitative PCR (qPCR). (B) Expression of the EBNA3A-repressed genes CXCL9 and CXCL10 were assessed by qPCR on the EBNA3A-KO and EBNA3A-REV (D1, D2, D3 and D4) cell lines as a control for the presence/absence of functional EBNA3A (C) Expression of EBNA3C-repressed genes ADAM28 and ADAMDEC1 were also determined by qPCR on the EBNA3C-conditional (p16-null cell lines) cultured with (+HT) or without 4HT (Washed) as a control for the functional EBNA3C. (TIF) [file ppat.1005031.s008.tif]

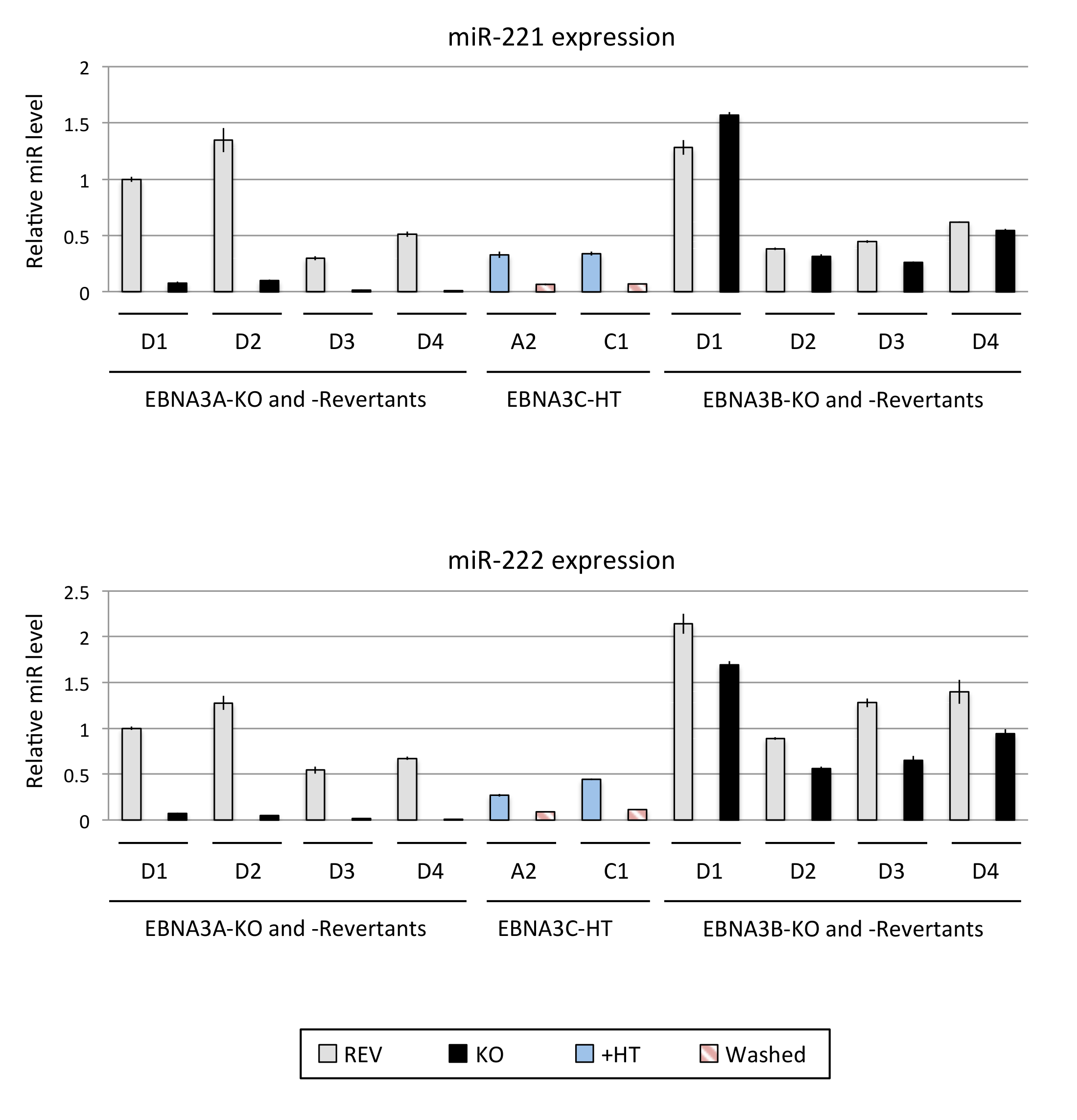

Supplement: S4 Fig — Levels of miR-221 and miR-222 were compared between LCL D1, D2, D3, D4 EBNA3A-KO and-REV, p16-null 3CHT A2 and C1 culture with (+HT) or without HT (Washed), LCL D1, D2, D3, D4 EBNA3B-KO and-REV. MiR expression was normalized to RNU6B and is shown relative to LCL D1 EBNA3A-REV. (TIF) [file ppat.1005031.s009.tif]

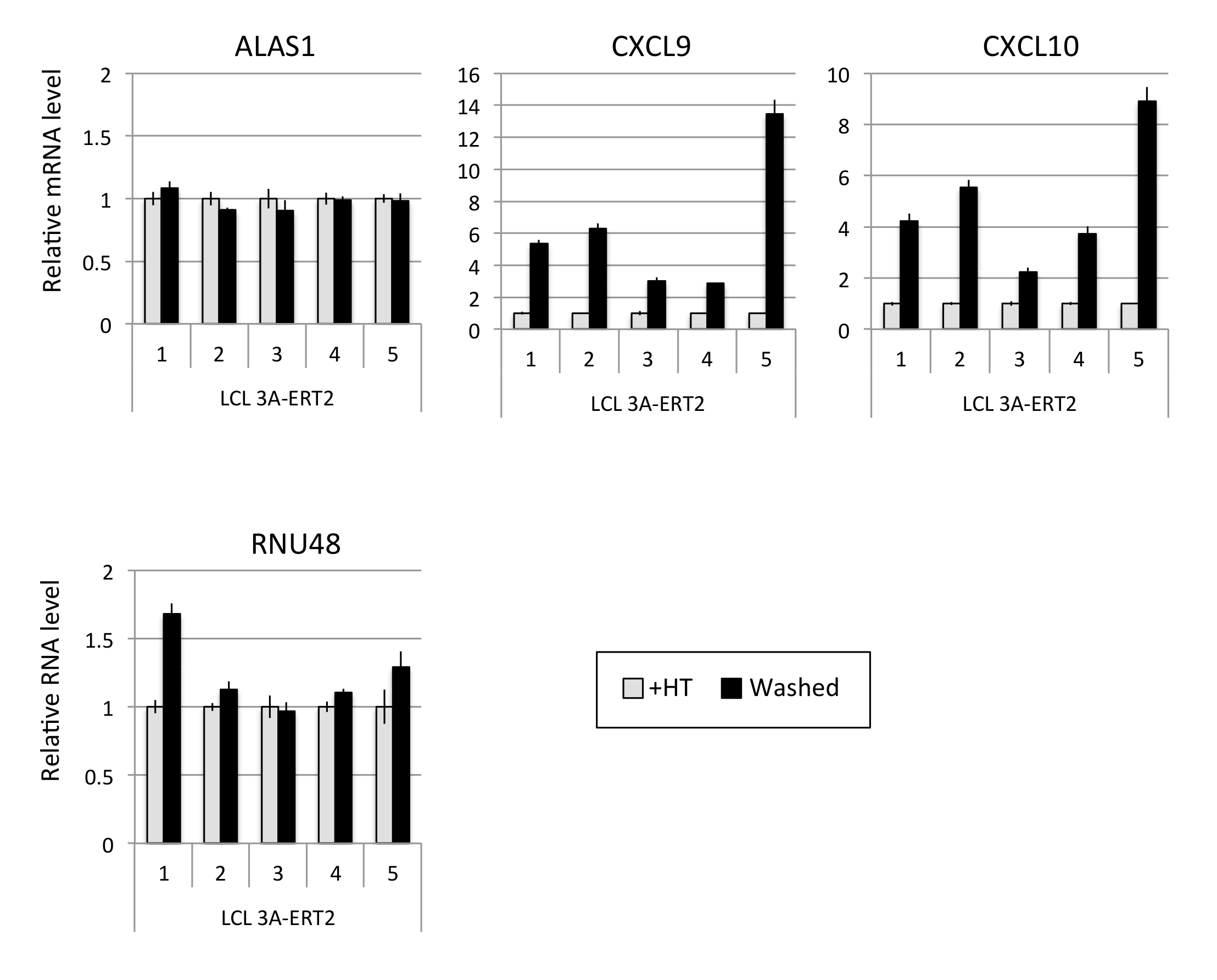

Supplement: S5 Fig — Expression levels of the well characterised EBNA3A repressed genes CXCL9 and CXCL10 were also determined as a control for the inactivation of EBN3A when 4HT was removed from the culture. Expression of control RNAs ALAS1 and RNU48 expression were also determined. (TIF) [file ppat.1005031.s010.tif]

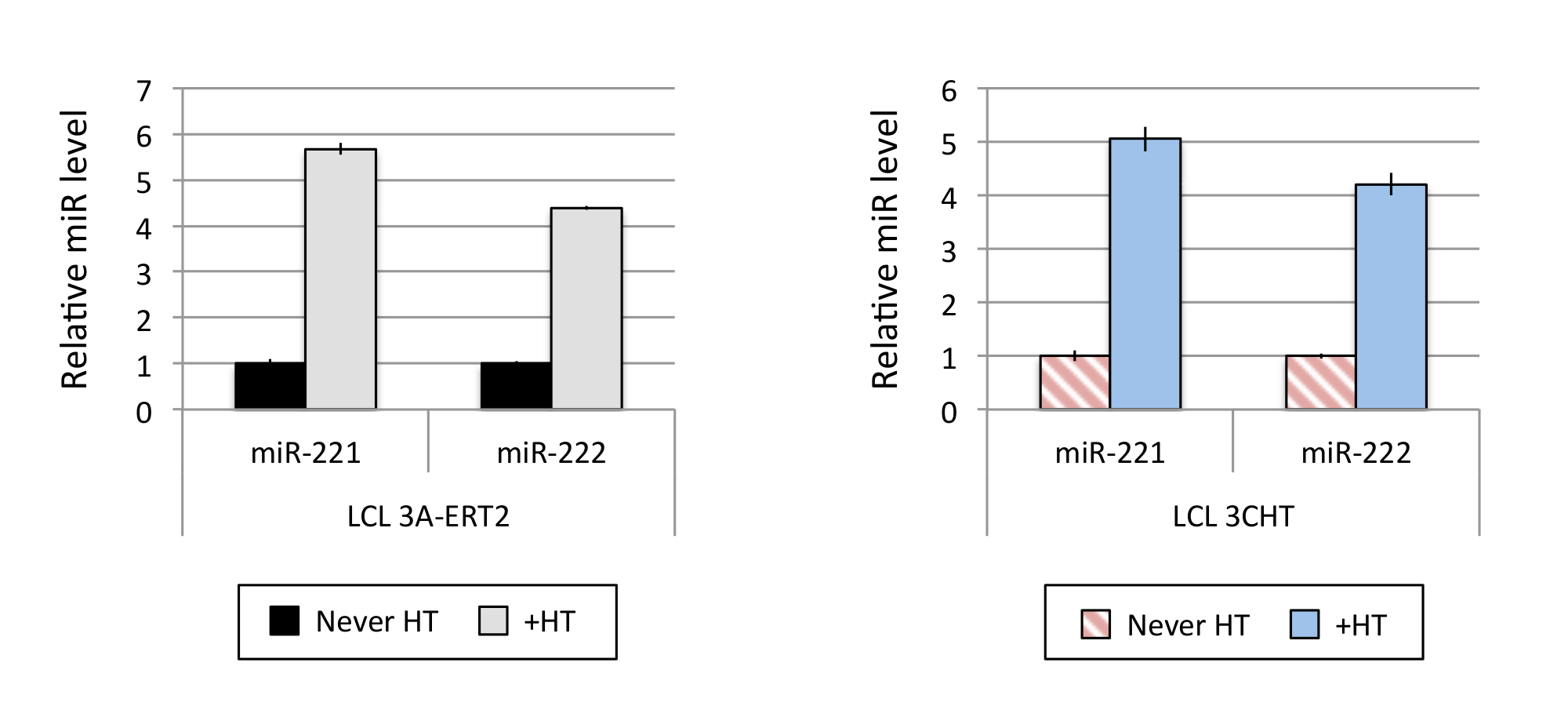

Supplement: S6 Fig — MiR-221 and miR-222 expression were determined by real time quantitative RT-PCR (qPCR) from EBNA3A-ERT2 LCLs established without the presence of 4HT (never HT) and 28 days after addition of 4HT to culture medium (+HT) and from p16-null LCL 3CHT also established without the presence of 4HT (never HT) or 30 days after 4HT (+HT) was added to culture medium. (TIF) [file ppat.1005031.s011.tif]

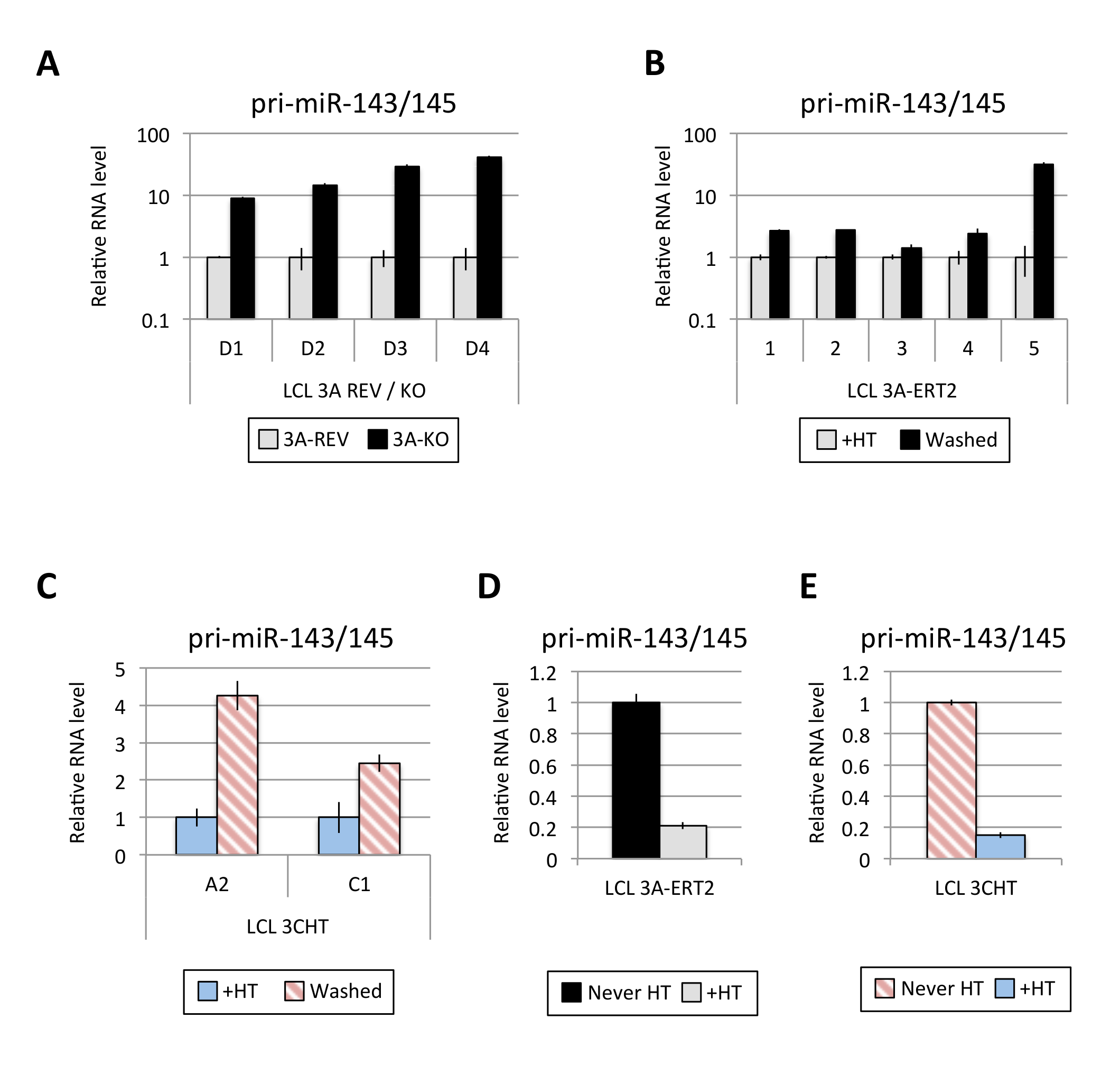

Supplement: S7 Fig — The expression level of the non-coding RNA precursor of miR-143/miR-145 was determined (A) in EBNA3A-KO and-REV LCLs; (B) in EBNA3A-ERT2 LCLs cultured with (+HT) or without 4HT (Washed); (C) in p16-null LCLs 3CHT with (+HT) or without 4HT (Washed); (D) in LCL EBNA3A-ERT2 (never HT) cultured for 28 days with (+HT) or without 4HT; (E) and p16-null LCL 3CHT (never HT) and cultured for 30 days with (+HT) or without 4HT. (TIF) [file ppat.1005031.s012.tif]

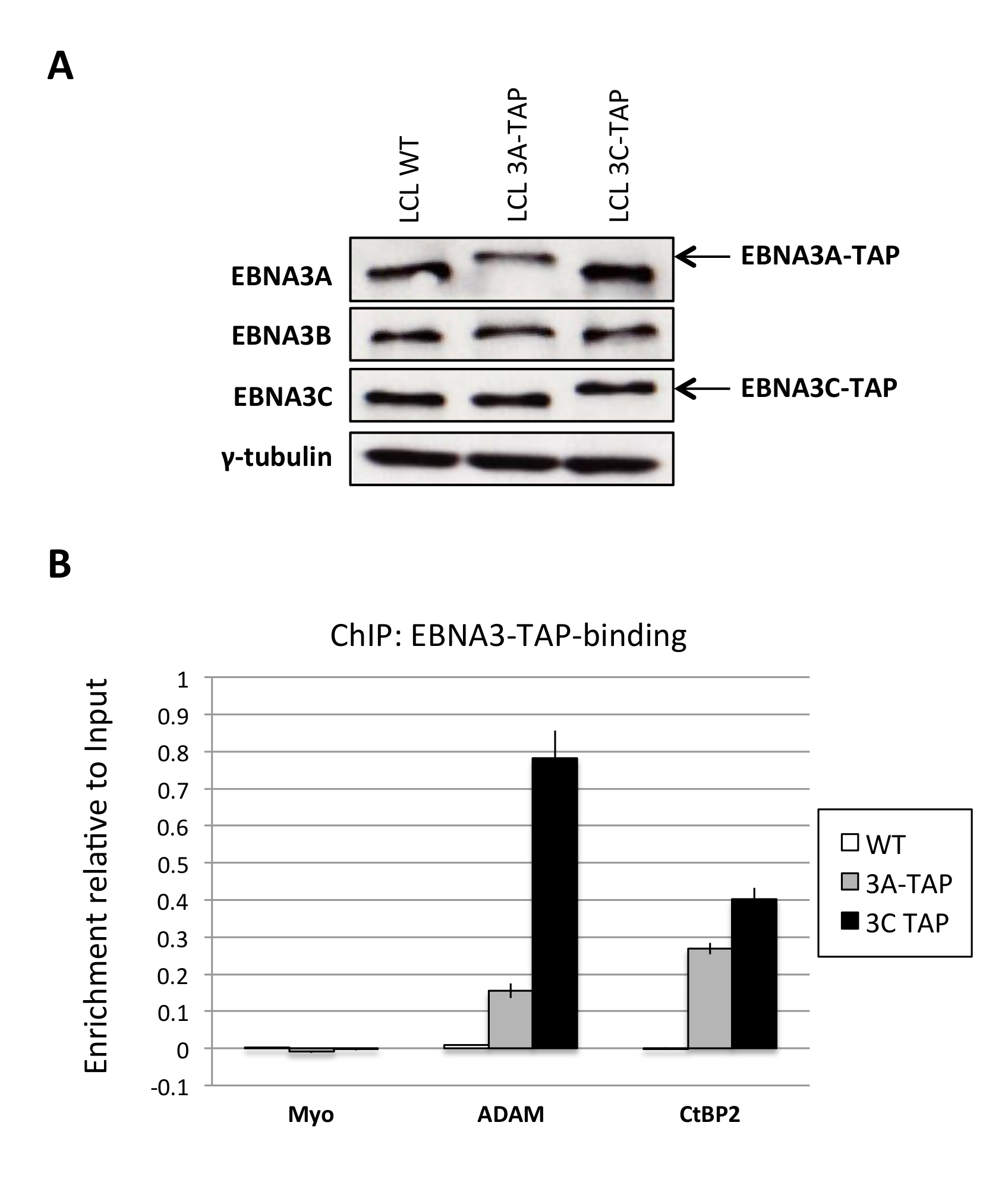

Supplement: S8 Fig — (A) EBNA3A-TAP, EBNA3B and EBNA3C-TAP expression in the cell lines LCL WT, LCL 3A-TAP, LCL 3C-TAP used in ChIP experiment was evaluated by Western blot. The blot was probed for γ-tubulin as a control for loading. (B). ChIP analysis using an anti-Flag antibody was performed as in Fig 7. Primers for the Myoglobin promoter (Myo) were used for qPCR as negative control, whereas primers for known EBNA3A/3C binding sites at the ADAM28/ADAMDEC1 intergenic enhancer (ADAM) and CtBP2 locus (CTBP2) were used as positive controls of EBNA3 binding. Values represent ratio of chromatin precipitated, after correction for IgG, relative to 2.5% of input. (TIF) [file ppat.1005031.s013.tif]

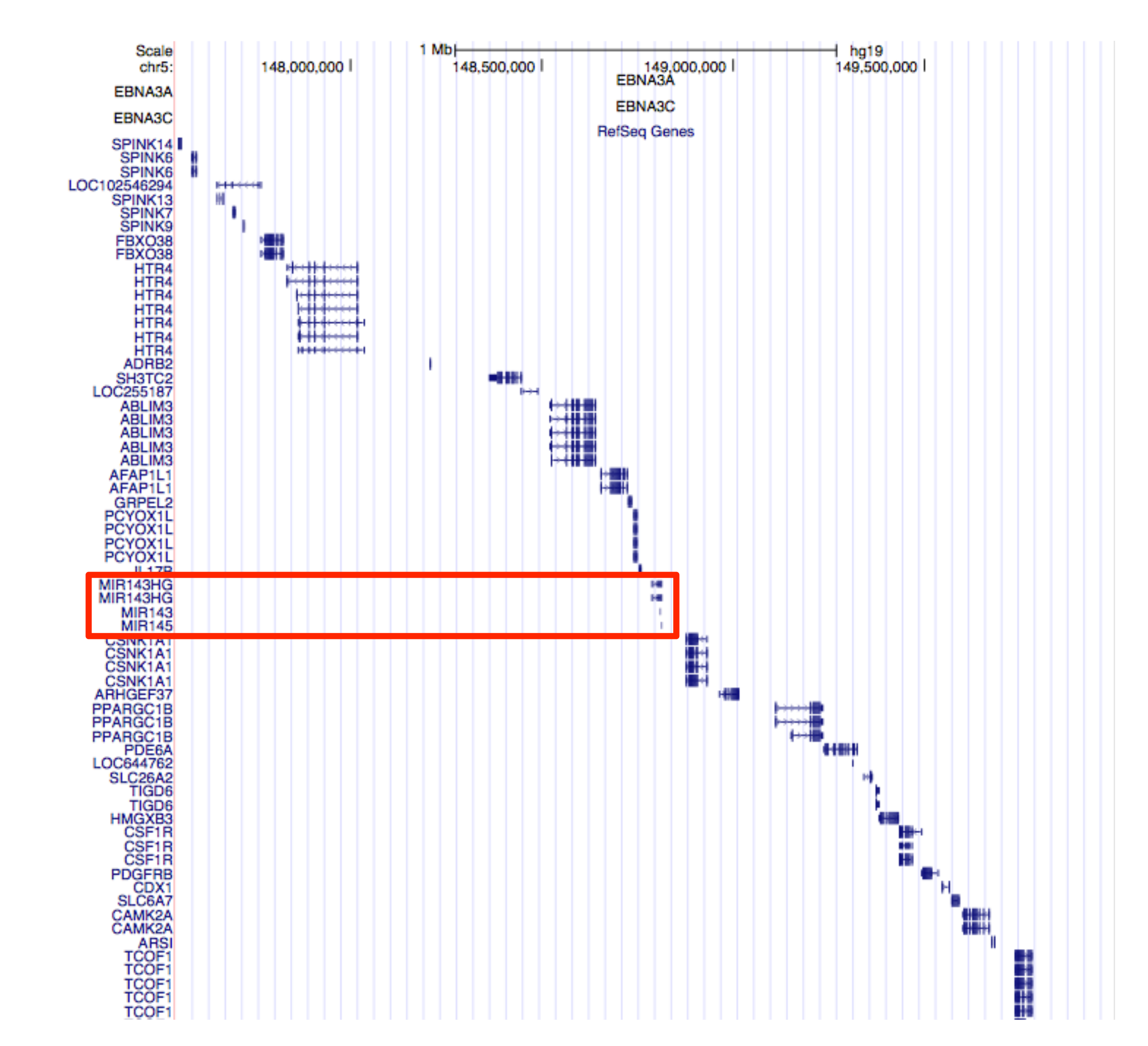

Supplement: S9 Fig — ChIP-seq data at the miR-143/miR-145 cluster genomic locus generated from LCL 3A-TAP and LCL 3C-TAP (Paschos et al., manuscript in preparation) were displayed using UCSC Genome Browser. The non-coding pri-miR-143/145 (called MIR143-HG in the genome browser) as well as miR-143/miR-145 are highlighted by inclusion in a red box. (TIF) [file ppat.1005031.s014.tif]

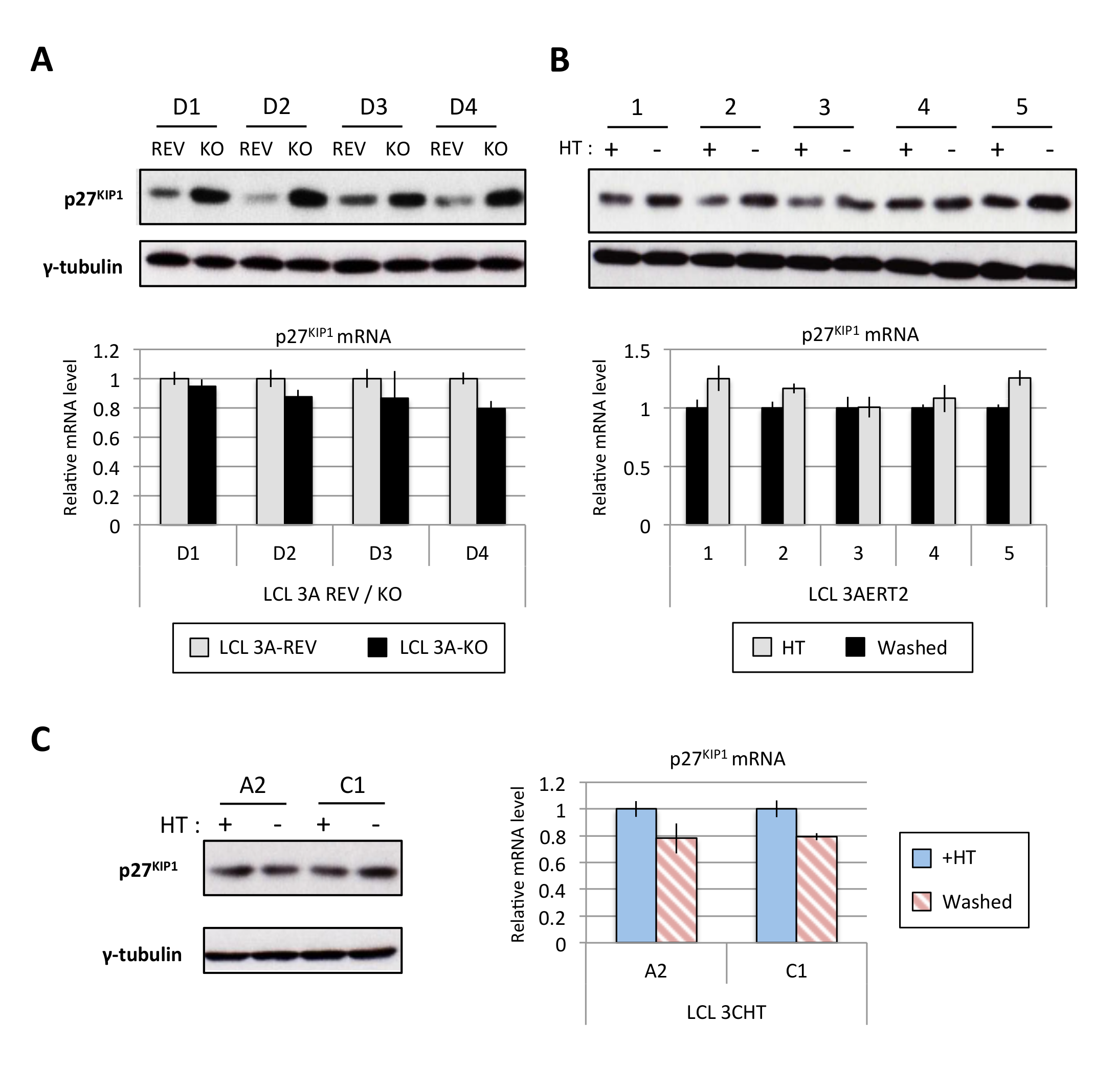

Supplement: S10 Fig — (A) Western blot showing the expression level of p27KIP1 from four EBNA3A-KO (KO) LCLs and their revertant equivalents (REV). Below the Western blot is the p27KIP1 mRNA expression level. (B) As in (A), but from five LCLs EBNA3A-ERT2 conditional cell lines cultured with (+) or without (-) 4HT for ~30 days. (C) Western blot showing the expression of p27KIP1 protein and qPCR showing the mRNA level corresponding to p27KIP1 from two (p16-null) LCL 3CHT cultured with (+) or without (-) 4HT. All the blots were probed for γ-tubulin as a control for loading. (TIF) [file ppat.1005031.s015.tif]

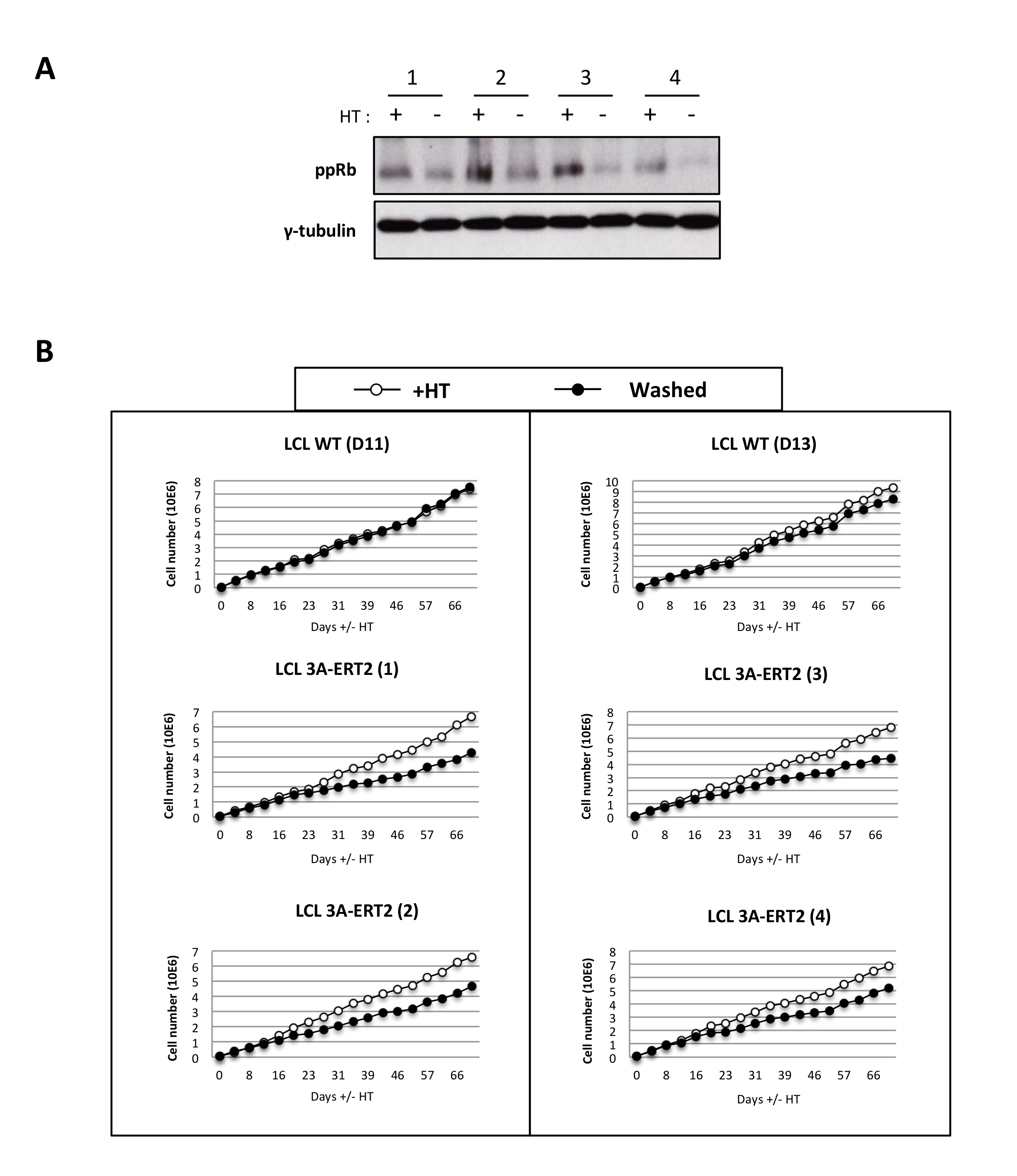

Supplement: S11 Fig — (A) Western blot showing the expression of hyperphosphorylated Rb (ppRb). γ-tubulin was used as a control for loading. (B) A comparison of the population growth rate between four 3A-ERT2 LCLs (line 1–2 being established from donor D11 and line 3–4 donor D13) cultured with (+HT) or without (Washed) 4HT for ~2 months was analysed by counting the number of viable cells every 2–3 days. Total cell numbers were plotted at each time point. As control, two wild-type LCLs from the same background as 3A-ERT2 LCLs (D11 and D13) were treated or not with HT. Data are representative of at least two independent experiments. (TIF) [file ppat.1005031.s016.tif]

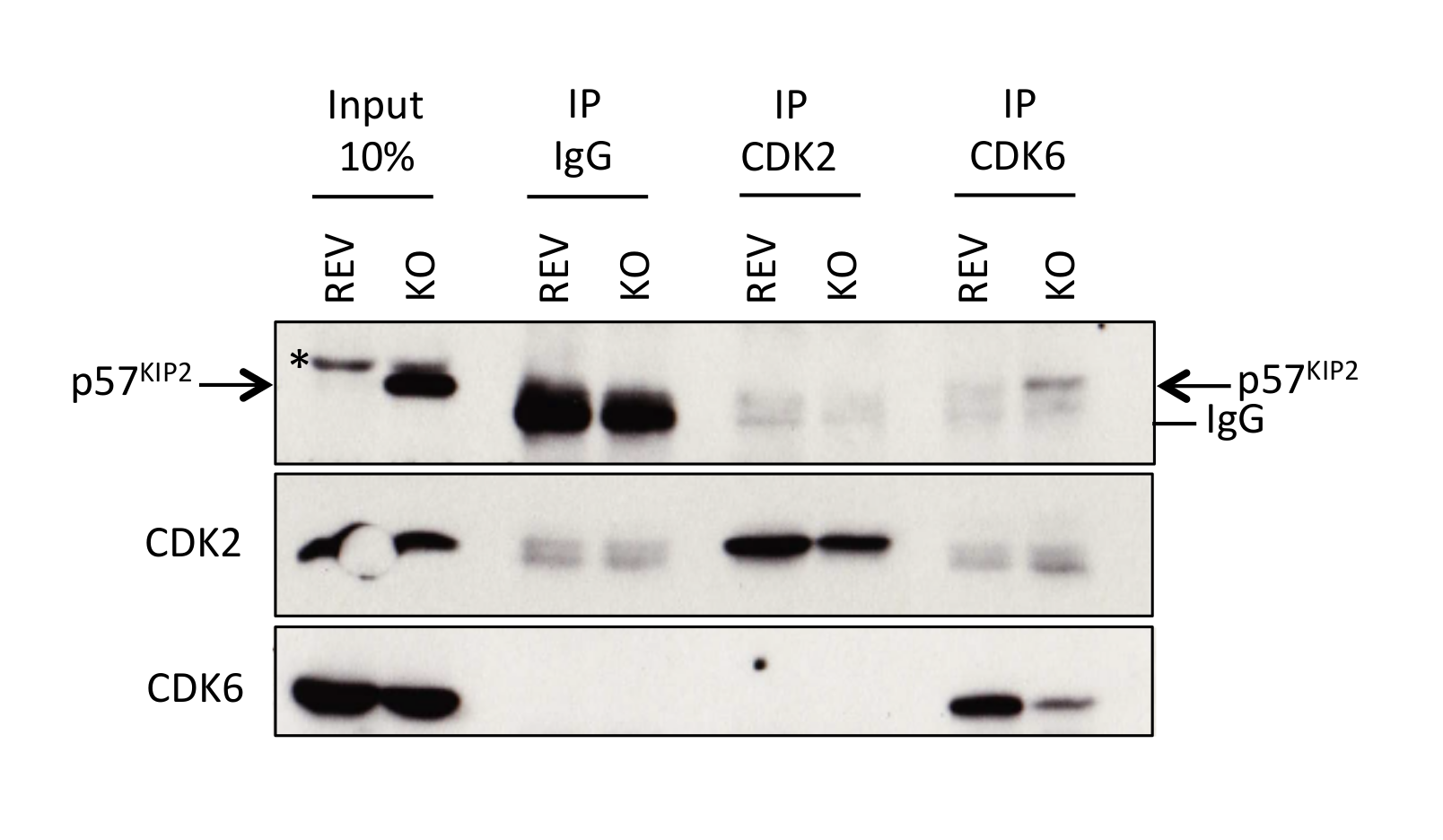

Supplement: S12 Fig — Immunoprecipitation was performed with mouse anti-CDK2 or anti-CDK6 antibodies on extracts from LCL D4 EBNA3A-REV and EBNA3A-KO. A large excess of mouse IgG was used as a control for non-specific binding and precipitates were compared to 10% input after Western blots were probed for p57KIP2, CDK2 or CDK6. p57KIP2 (arrowed) appears to precipitate with CDK6 but not CDK2. Immunoglobulin chains are indicated and the asterisk indicates an unidentified non-specific protein band. (TIF) [file ppat.1005031.s017.tif]

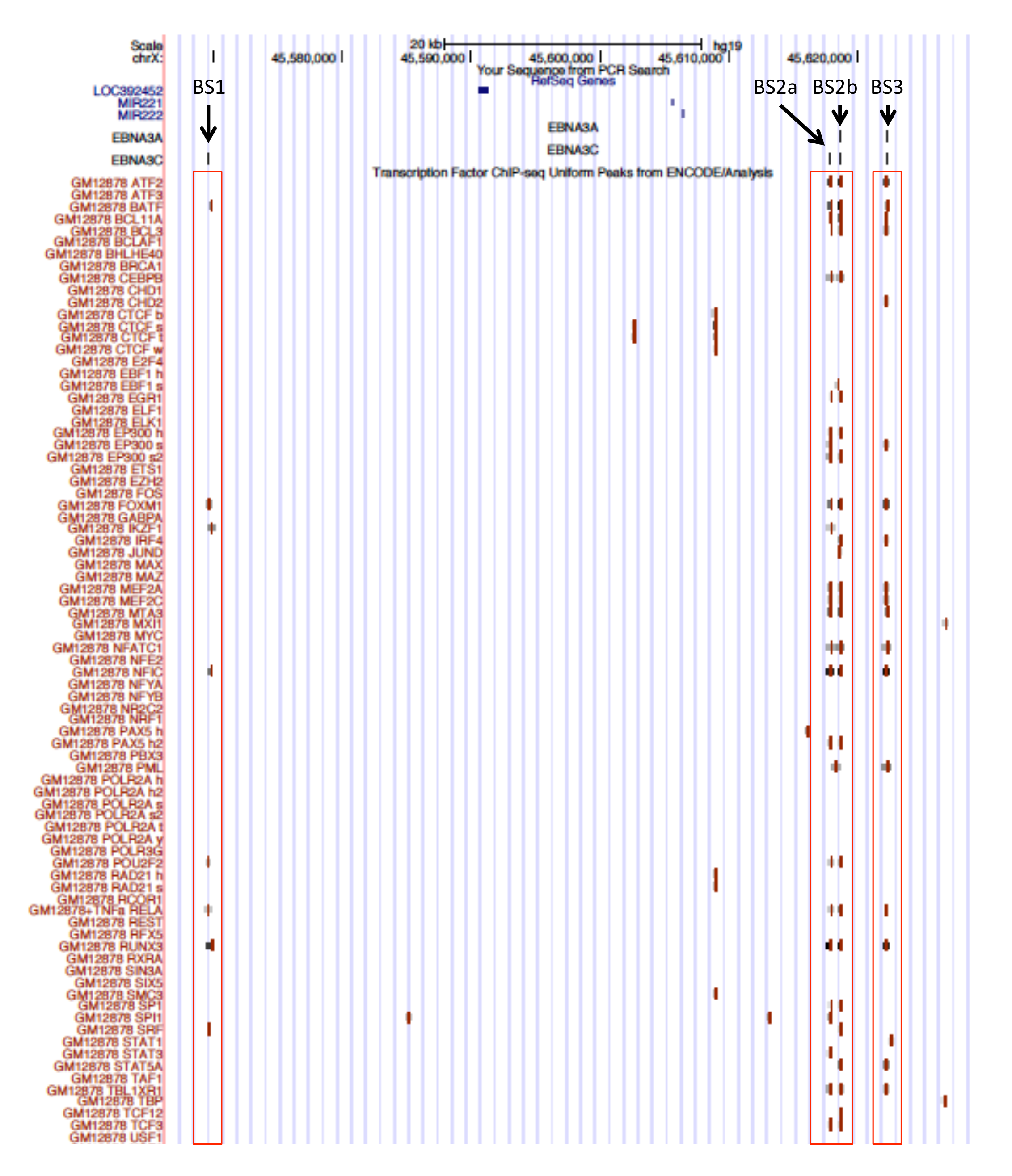

Supplement: S13 Fig — ENCODE GM12878 ChIP-seq data at the EBNA3A and EBNA3C binding sites BS1, BS2 (BS2a and BS2b) and BS3 showed multiple transcription factors also bind to those regions (displayed using UCSC Genome Browser). The three EBNA3s binding sites are highlighted by inclusion in a red box. (TIF) [file ppat.1005031.s018.tif]
